# Supplementary material for: Mitochondrial fusion and altered beta-oxidation drive muscle wasting in a Drosophila cachexia model
Source: EMBO Rep. 2024 Mar 1;25(4):15. doi: 10.1038/s44319-024-00102-z (PMC11014992; doi:10.1038/s44319-024-00102-z)
Supplement: Supplementary file 1 — Computer Code EV1 [file 44319_2024_102_MOESM1_ESM.zip › Computer Code EV1/computer macro 1.rtf]

Link to publications that use the data:
https://www.biorxiv.org/content/10.1101/2023.06.23.546217v1

Methods
The number of LDs present in the fly muscle was determined through the use of a macro in FIJI (Computer code EV 1). In brief, files were imported into FIJI, and a 200 x 200 pixel ROI was created on an 8-bit converted representative slice. The image was cropped to the ROI, then the “Auto-threshold” function was used to convert the image into binary. The “Analyse Particles” function using a size range of “0.00-10” was then used to count the number of LDs present in the image. This was then normalised to the size of the ROI in mm2.
The number of large extramyocellular LDs present in the mouse muscle was determined using a macro in FIJI (Computer code EV 1). In brief, files were imported into FIJI, a representative slice was converted to 8-bit. The “Auto-threshold” function was used to convert the image into binary. The “Analyse Particles” function using a size range of “2-infinity” was then used to count the number and size of extramyocellular LDs present in the image. The number of LDs was then normalised to the size of the ROI in mm2.
The number of intramyocellular LDs present in the mouse muscle was determined using a macro in FIJI (Computer code EV 1). In brief, files were imported into FIJI, and the “Auto-threshold” function was used to convert the image into binary. Five polygon ROIs that each encompassed the interior of a different myofiber was created on an 8-bit converted representative slice. The “Analyse Particles” function using a size range of “0.00-10” was then used to count the number and size of LDs present in the image. The number and size of LDs was averaged between the five myofibers for one image. The number of LDs was then normalised to the size of the ROI in mm2.
The proportion of mitochondrial sizes in the muscle was determined through applying a Log10 transformation to the list of areas outputted from the automated mitochondrial analysis pipeline (see above) to bring it closer to a normal distribution. The data was then binned into three sizes, those with a Log10 transformed value: X ≤ -0.5 (small), -0.5 < X ≤ 0 (medium), and X > 0 (large). The percentage of mitochondria in each of these categories was averaged across replicates, and the distribution of mitochondrial size across categories was compared via Chi-square test.
