# Supplementary material for: Mitochondrial fusion and altered beta-oxidation drive muscle wasting in a Drosophila cachexia model
Source: EMBO Rep. 2024 Mar 1;25(4):15. doi: 10.1038/s44319-024-00102-z (PMC11014992; doi:10.1038/s44319-024-00102-z)
Supplement: Supplementary file 2 — Computer Code EV2 [file 44319_2024_102_MOESM2_ESM.zip › Computer Code EV2/computer EV2.rtf]

Link to publications that use the data:
https://www.biorxiv.org/content/10.1101/2023.06.23.546217v1

Methods
The level of TMRE membrane potential was determined through the use of a macro in FIJI. In brief, files were imported into FIJI and split into MitoTrackerTM Green and TMRE channels. A max intensity z-projection of the MitoTrackerTM Green channel was converted to 8-bit, smoothed using the “Gaussian Blur” and “Remove Outliers” functions, and a square ROI was drawn around the muscle fillet. The image was then converted to a binary mask using the “Auto Threshold” function using the “IsoData” method. A selection was then created around all the thresholded MitoTrackerTM Green area, and this area was measured. A max intensity z-projection of the TMRE channel was then processed in the same way as the MitoTrackerTM Green channel. The final result was an area measurement for total mitochondria via MitoTrackerTM Green and active mitochondria via TMRE. The total area of TMRE was divided by the total area of MitoTrackerTM Green, to give a percentage value of how many total mitochondria are active. 
