# Supplementary figures and images for: Mitochondrial fusion and altered beta-oxidation drive muscle wasting in a Drosophila cachexia model
Source: EMBO Rep. 2024 Mar 1;25(4):15. doi: 10.1038/s44319-024-00102-z (PMC11014992; doi:10.1038/s44319-024-00102-z)

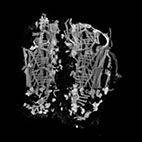

Supplement: Supplementary file 3 — Source Data Fig. 1 [file 44319_2024_102_MOESM3_ESM.zip › Figure 1 uploaded/Figure 1N.jpg]

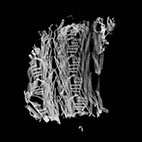

Supplement: Supplementary file 3 — Source Data Fig. 1 [file 44319_2024_102_MOESM3_ESM.zip › Figure 1 uploaded/Figure 1O.jpg]

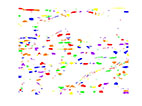

Supplement: Supplementary file 3 — Source Data Fig. 1 [file 44319_2024_102_MOESM3_ESM.zip › Figure 1 uploaded/Figure 1H.jpg]

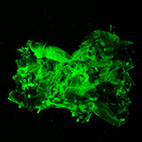

Supplement: Supplementary file 3 — Source Data Fig. 1 [file 44319_2024_102_MOESM3_ESM.zip › Figure 1 uploaded/Figure 1K.jpg]

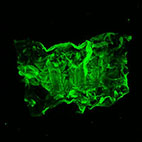

Supplement: Supplementary file 3 — Source Data Fig. 1 [file 44319_2024_102_MOESM3_ESM.zip › Figure 1 uploaded/Figure 1J.jpg]

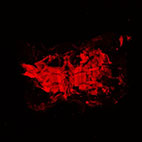

Supplement: Supplementary file 3 — Source Data Fig. 1 [file 44319_2024_102_MOESM3_ESM.zip › Figure 1 uploaded/Figure 1J'.jpg]

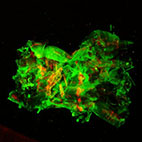

Supplement: Supplementary file 3 — Source Data Fig. 1 [file 44319_2024_102_MOESM3_ESM.zip › Figure 1 uploaded/Figure 1K''.jpg]

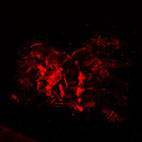

Supplement: Supplementary file 3 — Source Data Fig. 1 [file 44319_2024_102_MOESM3_ESM.zip › Figure 1 uploaded/Figure 1K'.jpg]

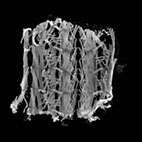

Supplement: Supplementary file 3 — Source Data Fig. 1 [file 44319_2024_102_MOESM3_ESM.zip › Figure 1 uploaded/Figure 1P.jpg]

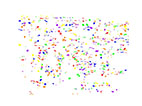

Supplement: Supplementary file 3 — Source Data Fig. 1 [file 44319_2024_102_MOESM3_ESM.zip › Figure 1 uploaded/Figure 1G.jpg]

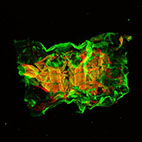

Supplement: Supplementary file 3 — Source Data Fig. 1 [file 44319_2024_102_MOESM3_ESM.zip › Figure 1 uploaded/Figure 1J''.jpg]

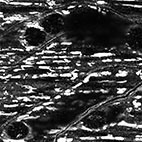

Supplement: Supplementary file 3 — Source Data Fig. 1 [file 44319_2024_102_MOESM3_ESM.zip › Figure 1 uploaded/Figure 1F.jpg]

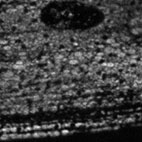

Supplement: Supplementary file 3 — Source Data Fig. 1 [file 44319_2024_102_MOESM3_ESM.zip › Figure 1 uploaded/Figure 1S.jpg]

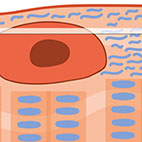

Supplement: Supplementary file 3 — Source Data Fig. 1 [file 44319_2024_102_MOESM3_ESM.zip › Figure 1 uploaded/Figure 1D.jpg]

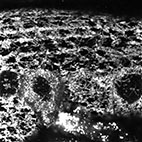

Supplement: Supplementary file 3 — Source Data Fig. 1 [file 44319_2024_102_MOESM3_ESM.zip › Figure 1 uploaded/Figure 1E.jpg]

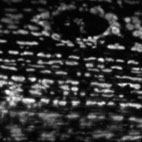

Supplement: Supplementary file 3 — Source Data Fig. 1 [file 44319_2024_102_MOESM3_ESM.zip › Figure 1 uploaded/Figure 1R.jpg]

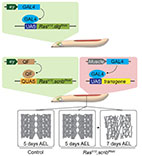

Supplement: Supplementary file 3 — Source Data Fig. 1 [file 44319_2024_102_MOESM3_ESM.zip › Figure 1 uploaded/Figure 1A.jpg]

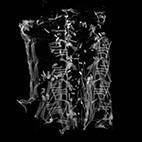

Supplement: Supplementary file 3 — Source Data Fig. 1 [file 44319_2024_102_MOESM3_ESM.zip › Figure 1 uploaded/Figure 1V.jpg]

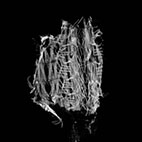

Supplement: Supplementary file 3 — Source Data Fig. 1 [file 44319_2024_102_MOESM3_ESM.zip › Figure 1 uploaded/Figure 1W.jpg]

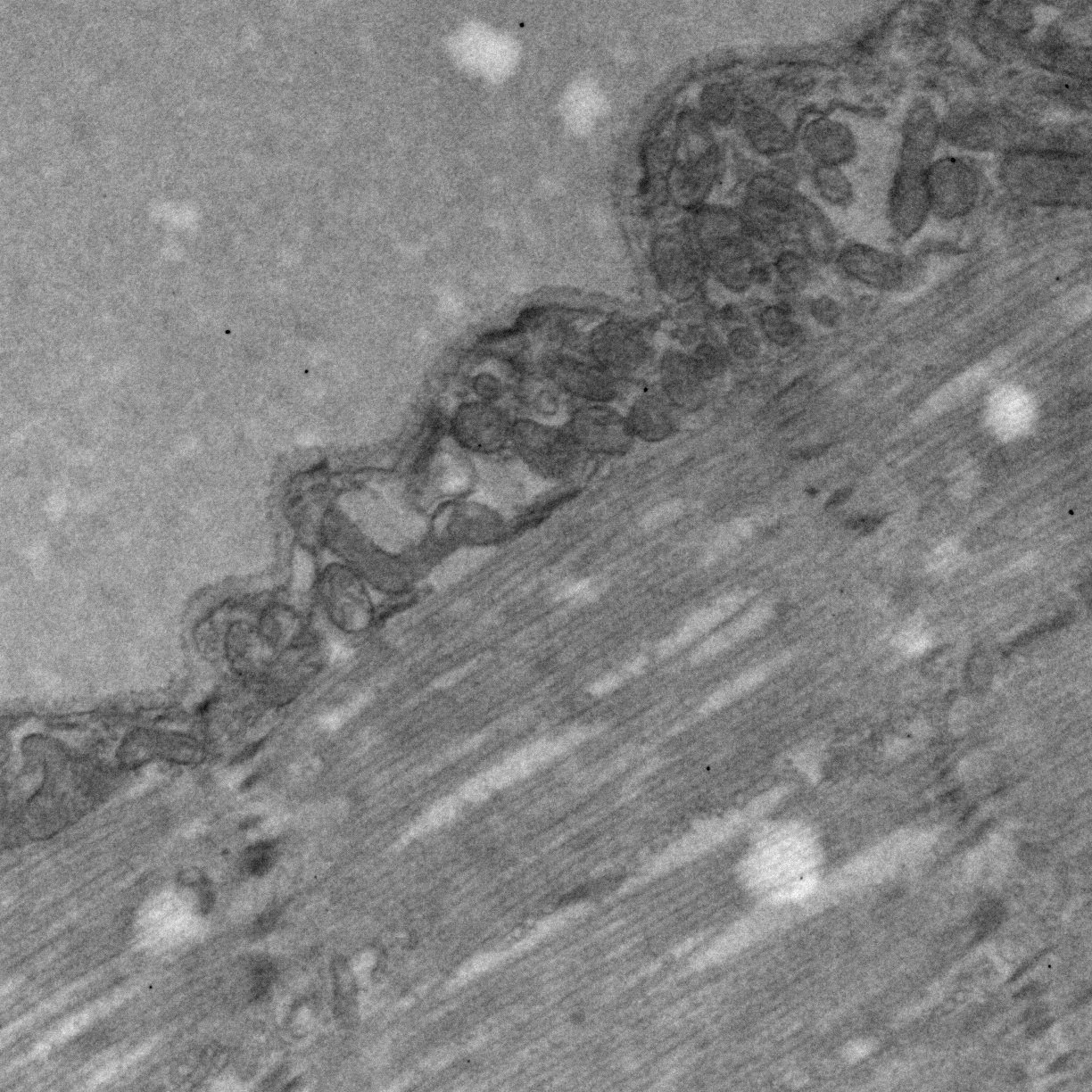

Supplement: Supplementary file 3 — Source Data Fig. 1 [file 44319_2024_102_MOESM3_ESM.zip › Figure 1 uploaded/Figure 1B.jpg]

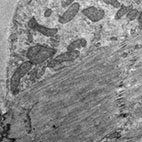

Supplement: Supplementary file 3 — Source Data Fig. 1 [file 44319_2024_102_MOESM3_ESM.zip › Figure 1 uploaded/Figure 1C.jpg]

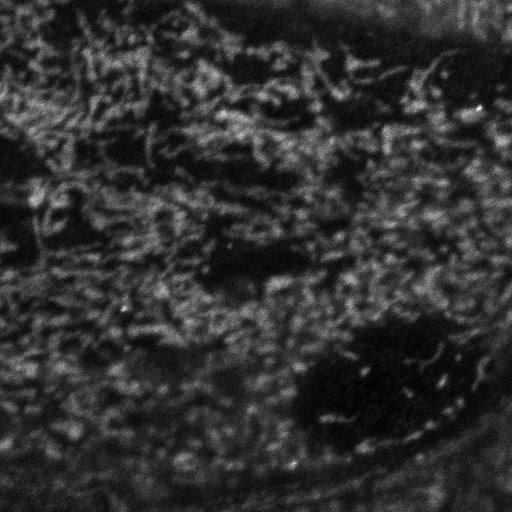

Supplement: Supplementary file 4 — Source Data Fig. 2 [file 44319_2024_102_MOESM4_ESM.zip › Figure 2A.jpg]

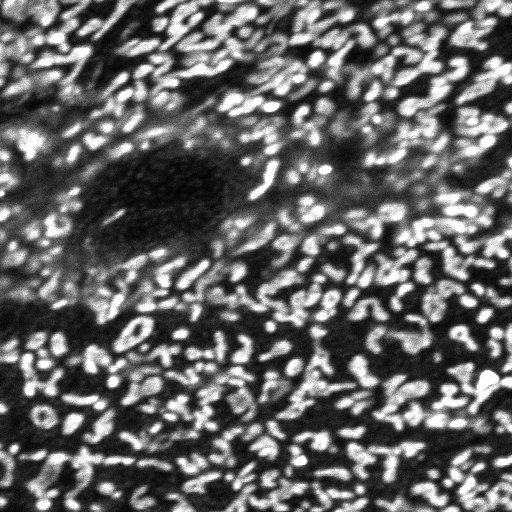

Supplement: Supplementary file 4 — Source Data Fig. 2 [file 44319_2024_102_MOESM4_ESM.zip › Figure 2B.jpg]

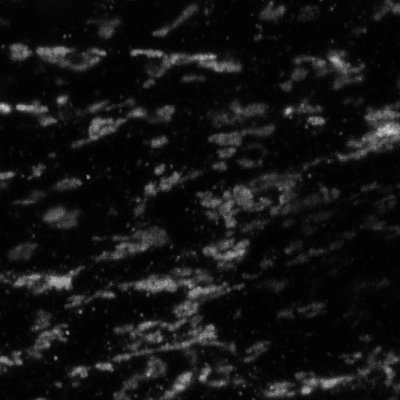

Supplement: Supplementary file 4 — Source Data Fig. 2 [file 44319_2024_102_MOESM4_ESM.zip › Figure 2D.jpg]

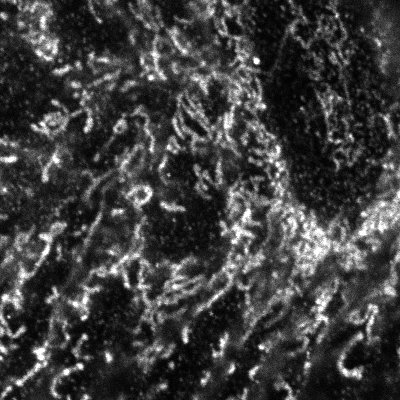

Supplement: Supplementary file 4 — Source Data Fig. 2 [file 44319_2024_102_MOESM4_ESM.zip › Figure 2E.jpg]

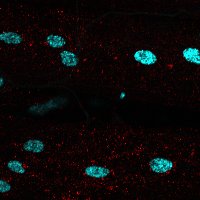

Supplement: Supplementary file 4 — Source Data Fig. 2 [file 44319_2024_102_MOESM4_ESM.zip › Figure 2H''.jpg]

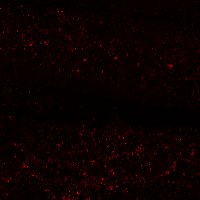

Supplement: Supplementary file 4 — Source Data Fig. 2 [file 44319_2024_102_MOESM4_ESM.zip › Figure 2H'.jpg]

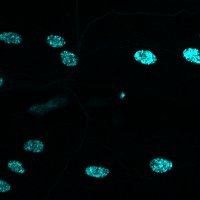

Supplement: Supplementary file 4 — Source Data Fig. 2 [file 44319_2024_102_MOESM4_ESM.zip › Figure 2H.jpg]

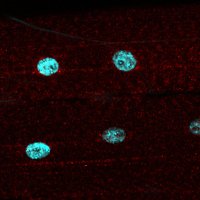

Supplement: Supplementary file 4 — Source Data Fig. 2 [file 44319_2024_102_MOESM4_ESM.zip › Figure 2I''.jpg]

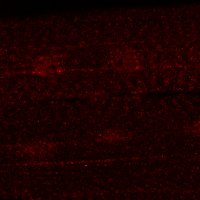

Supplement: Supplementary file 4 — Source Data Fig. 2 [file 44319_2024_102_MOESM4_ESM.zip › Figure 2I'.jpg]

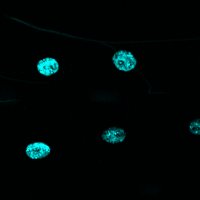

Supplement: Supplementary file 4 — Source Data Fig. 2 [file 44319_2024_102_MOESM4_ESM.zip › Figure 2I.jpg]

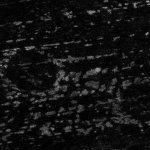

Supplement: Supplementary file 4 — Source Data Fig. 2 [file 44319_2024_102_MOESM4_ESM.zip › Figure 2L.jpg]

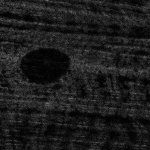

Supplement: Supplementary file 4 — Source Data Fig. 2 [file 44319_2024_102_MOESM4_ESM.zip › Figure 2M.jpg]

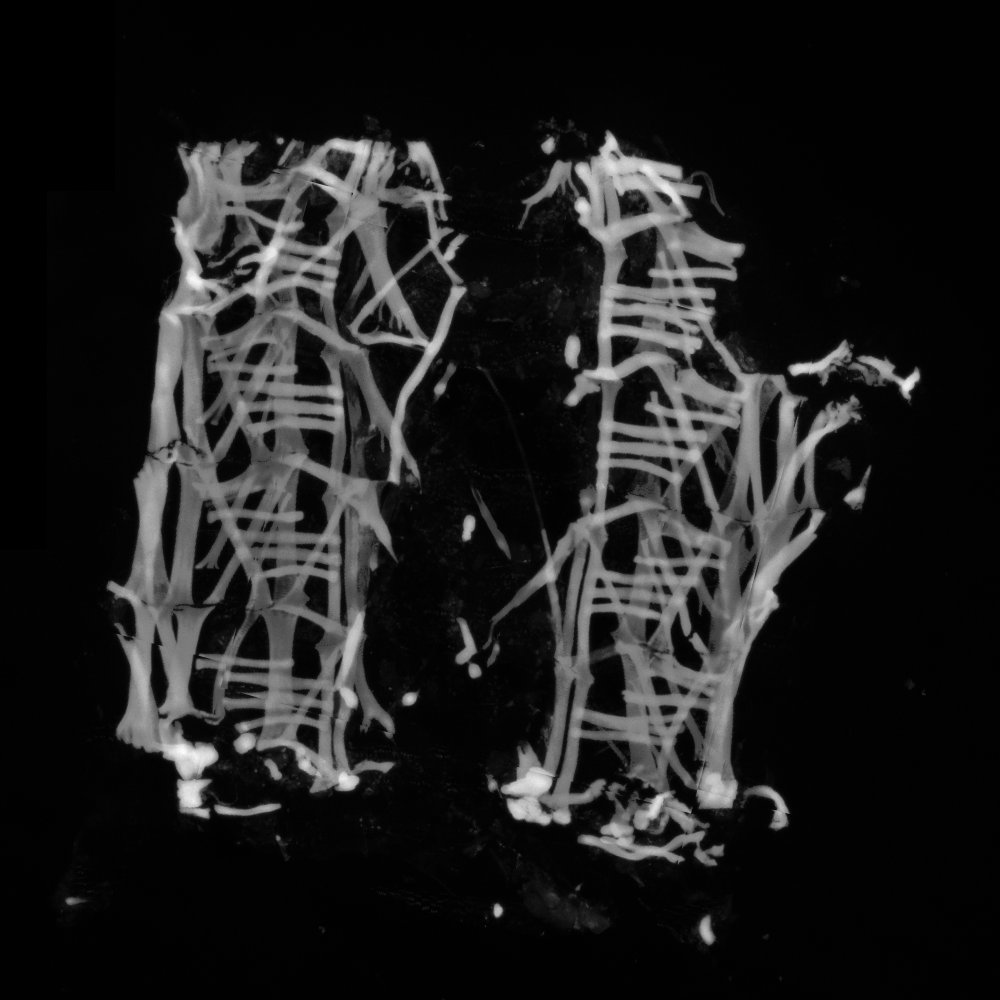

Supplement: Supplementary file 4 — Source Data Fig. 2 [file 44319_2024_102_MOESM4_ESM.zip › Figure 2O.jpg]

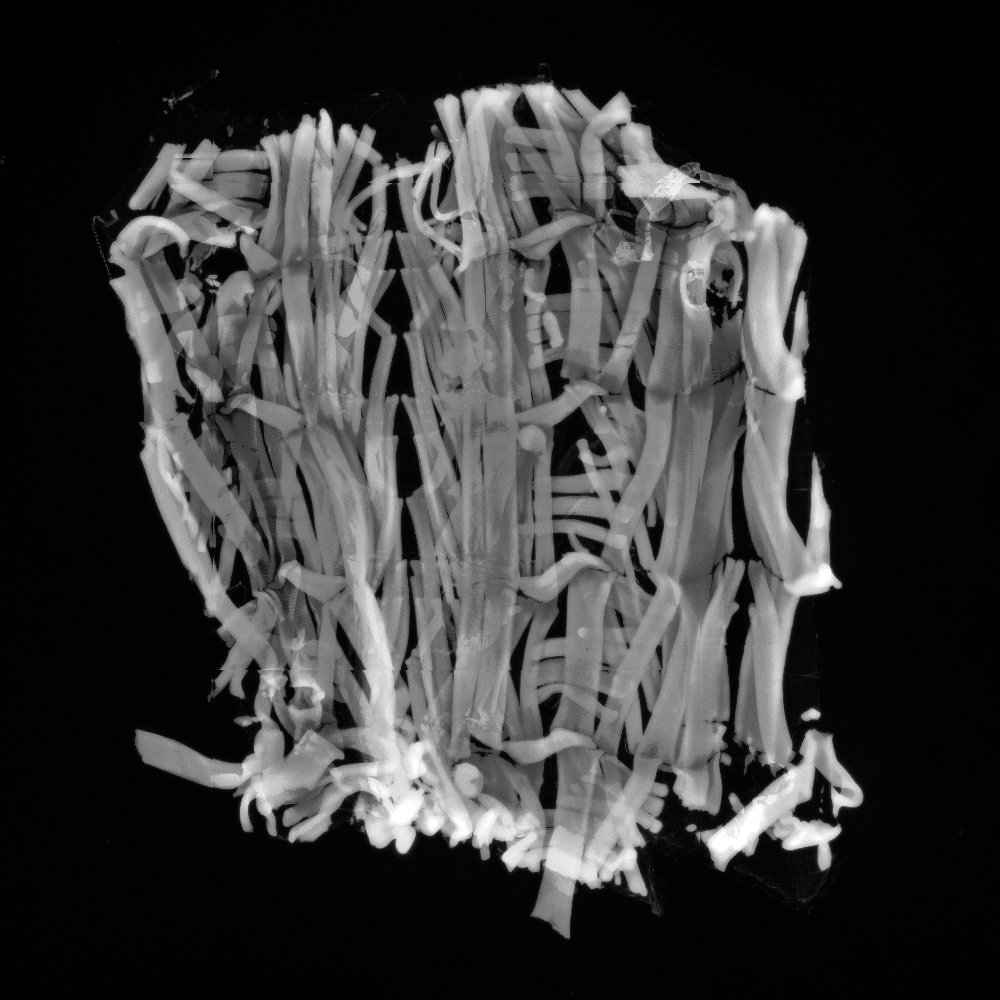

Supplement: Supplementary file 4 — Source Data Fig. 2 [file 44319_2024_102_MOESM4_ESM.zip › Figure 2P.jpg]

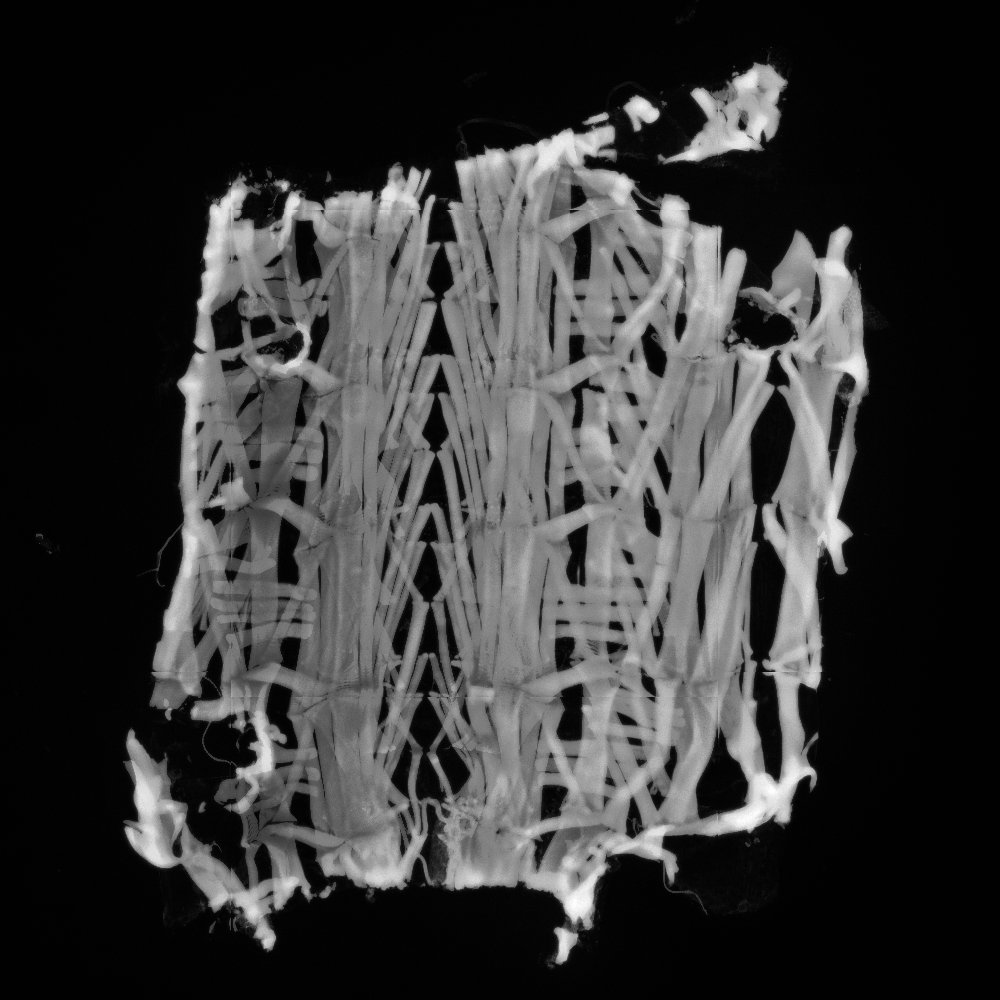

Supplement: Supplementary file 4 — Source Data Fig. 2 [file 44319_2024_102_MOESM4_ESM.zip › Figure 2Q.jpg]

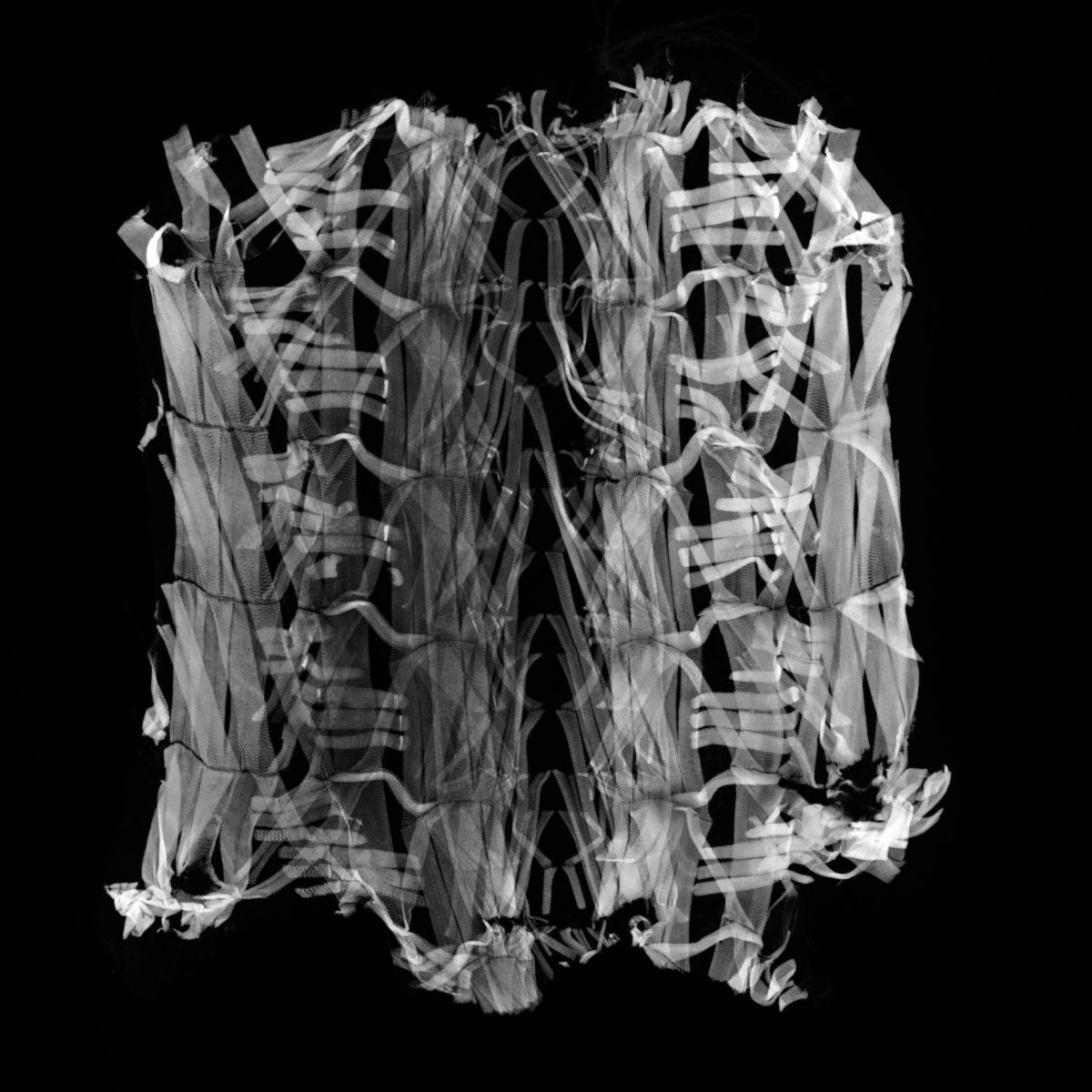

Supplement: Supplementary file 4 — Source Data Fig. 2 [file 44319_2024_102_MOESM4_ESM.zip › Figure 2T.jpg]

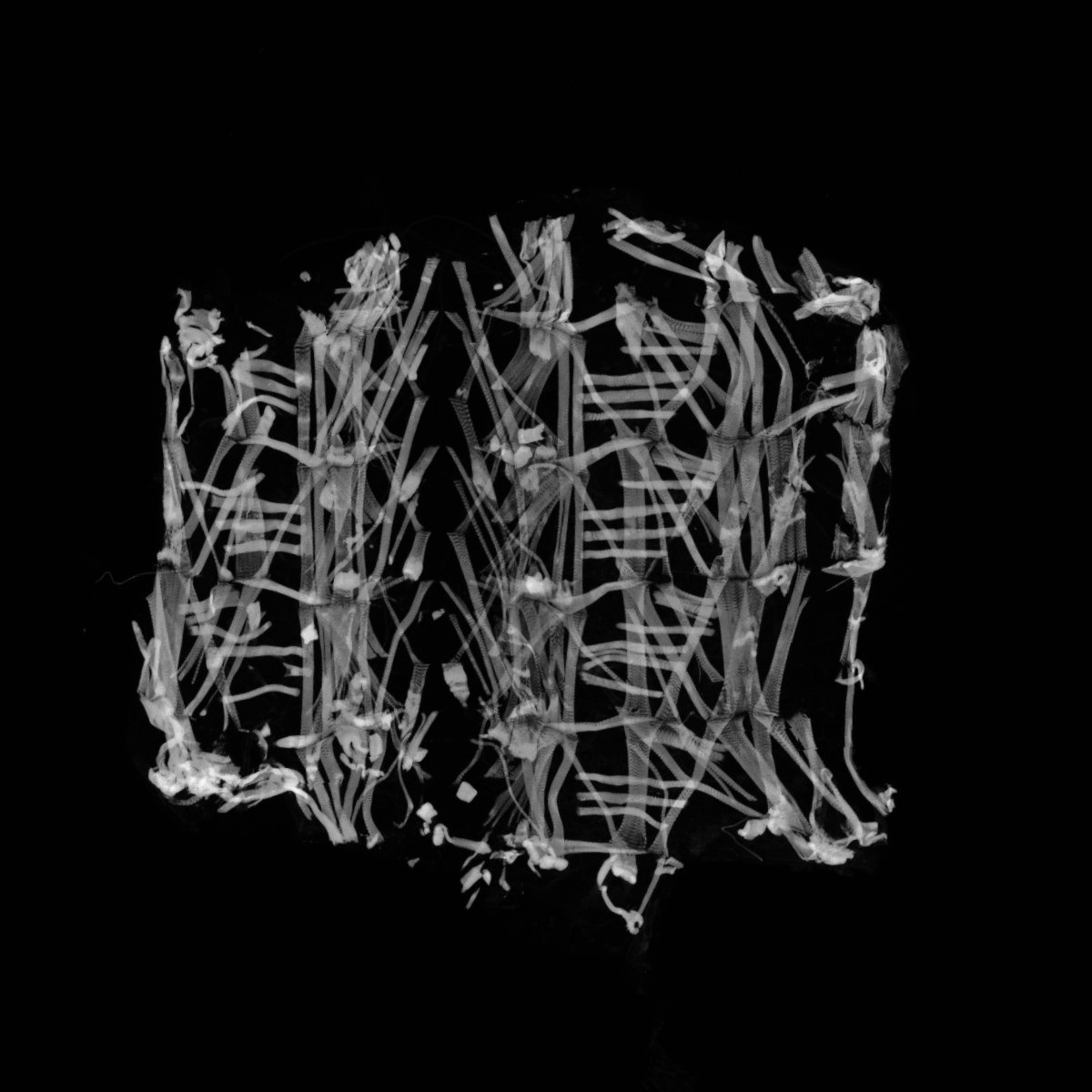

Supplement: Supplementary file 4 — Source Data Fig. 2 [file 44319_2024_102_MOESM4_ESM.zip › Figure 2U.jpg]

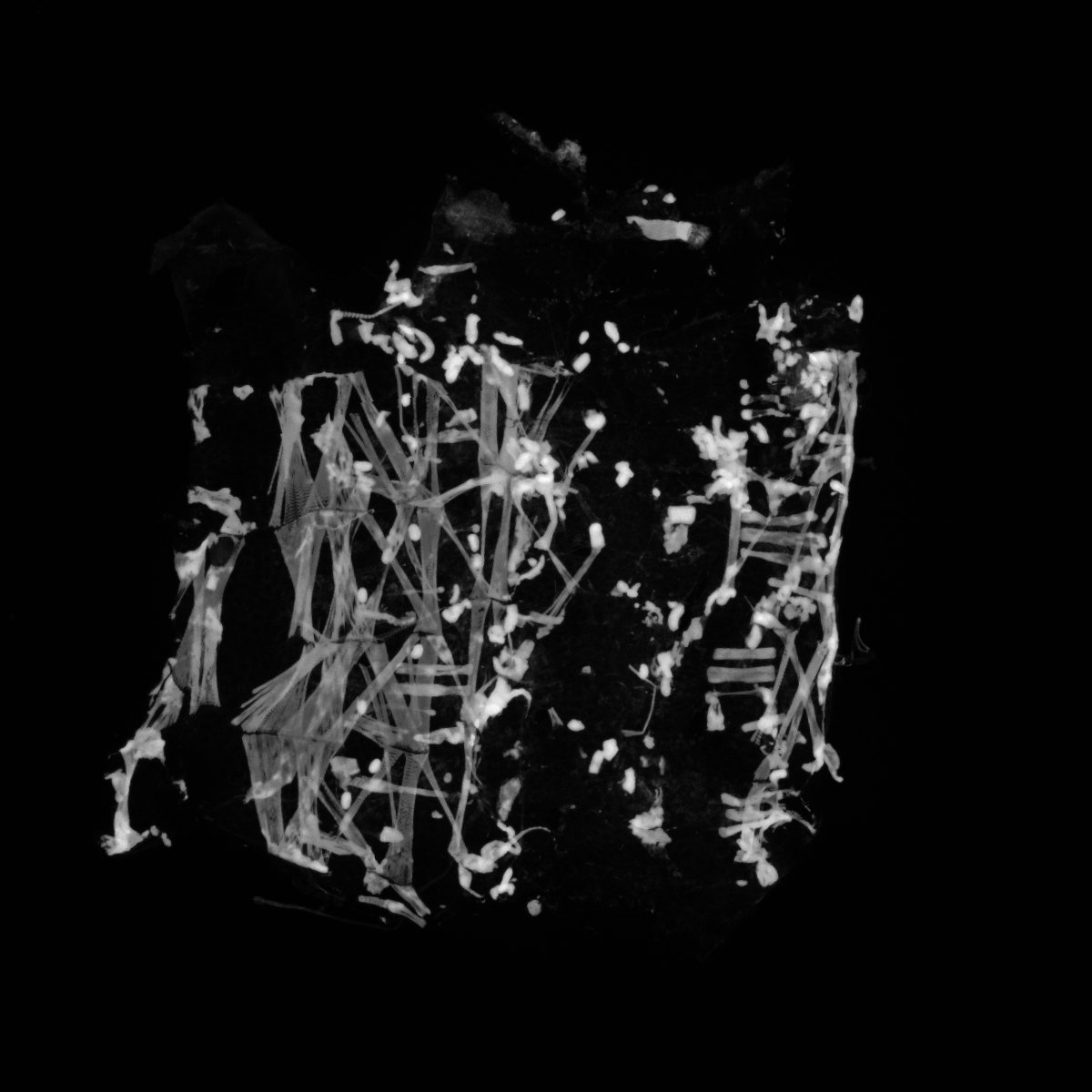

Supplement: Supplementary file 4 — Source Data Fig. 2 [file 44319_2024_102_MOESM4_ESM.zip › Figure 2V.jpg]

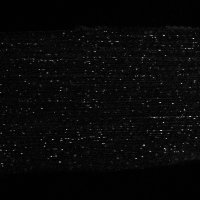

Supplement: Supplementary file 5 — Source Data Fig. 3 [file 44319_2024_102_MOESM5_ESM.zip › Figure 3A.jpg]

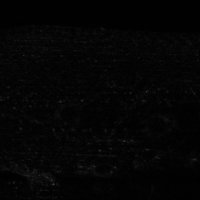

Supplement: Supplementary file 5 — Source Data Fig. 3 [file 44319_2024_102_MOESM5_ESM.zip › Figure 3B.jpg]

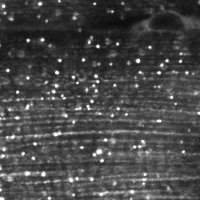

Supplement: Supplementary file 5 — Source Data Fig. 3 [file 44319_2024_102_MOESM5_ESM.zip › Figure 3D.jpg]

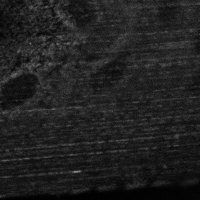

Supplement: Supplementary file 5 — Source Data Fig. 3 [file 44319_2024_102_MOESM5_ESM.zip › Figure 3E.jpg]

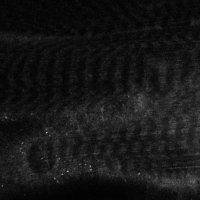

Supplement: Supplementary file 5 — Source Data Fig. 3 [file 44319_2024_102_MOESM5_ESM.zip › Figure 3G.jpg]

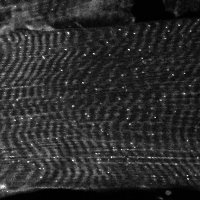

Supplement: Supplementary file 5 — Source Data Fig. 3 [file 44319_2024_102_MOESM5_ESM.zip › Figure 3H.jpg]

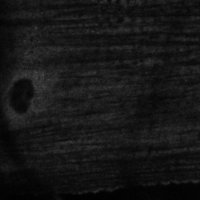

Supplement: Supplementary file 5 — Source Data Fig. 3 [file 44319_2024_102_MOESM5_ESM.zip › Figure 3J.jpg]

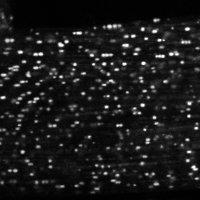

Supplement: Supplementary file 5 — Source Data Fig. 3 [file 44319_2024_102_MOESM5_ESM.zip › Figure 3K.jpg]

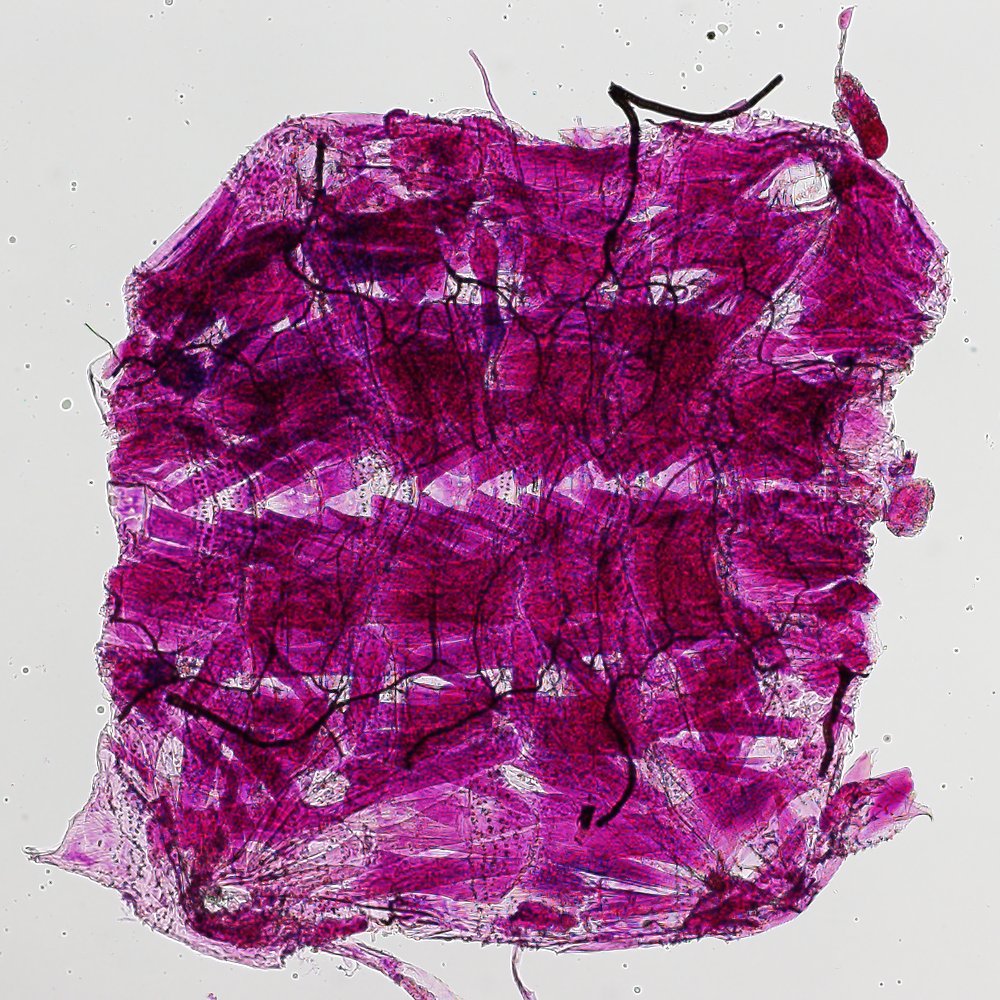

Supplement: Supplementary file 5 — Source Data Fig. 3 [file 44319_2024_102_MOESM5_ESM.zip › Figure 3M.jpg]

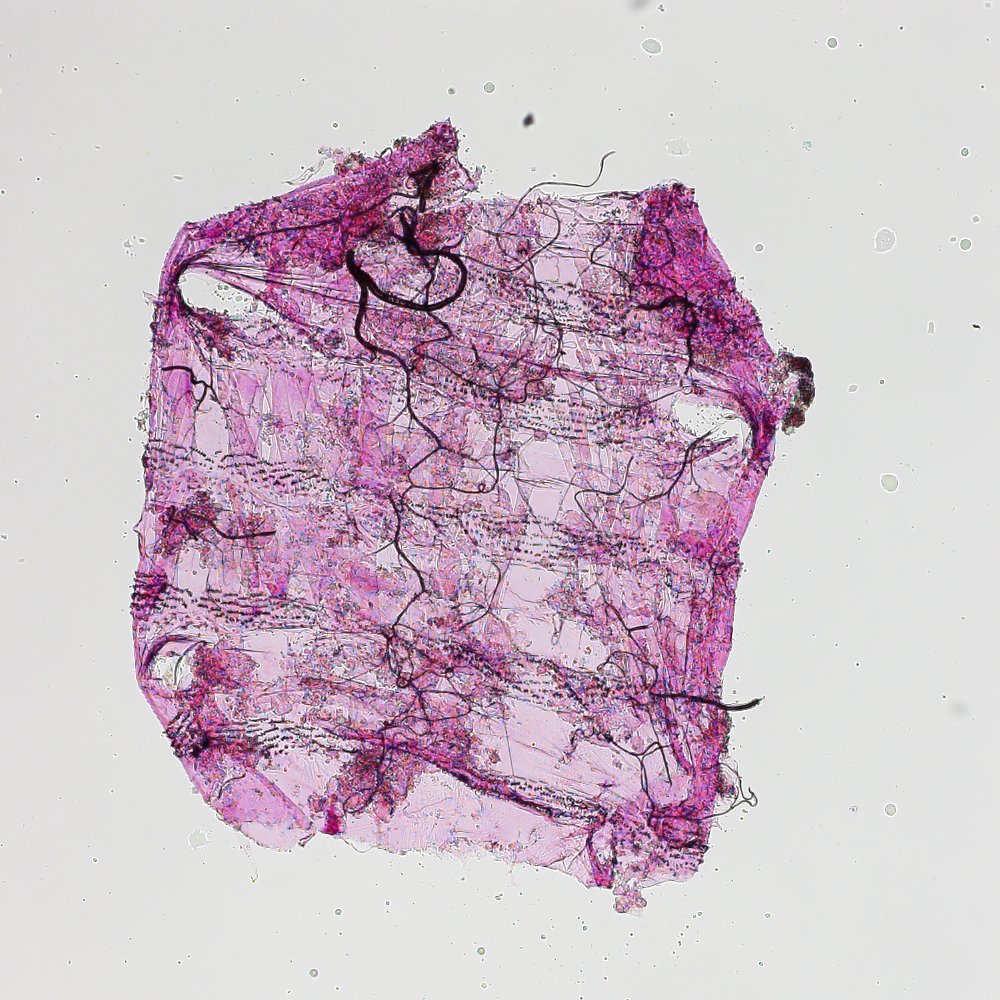

Supplement: Supplementary file 5 — Source Data Fig. 3 [file 44319_2024_102_MOESM5_ESM.zip › Figure 3N.jpg]

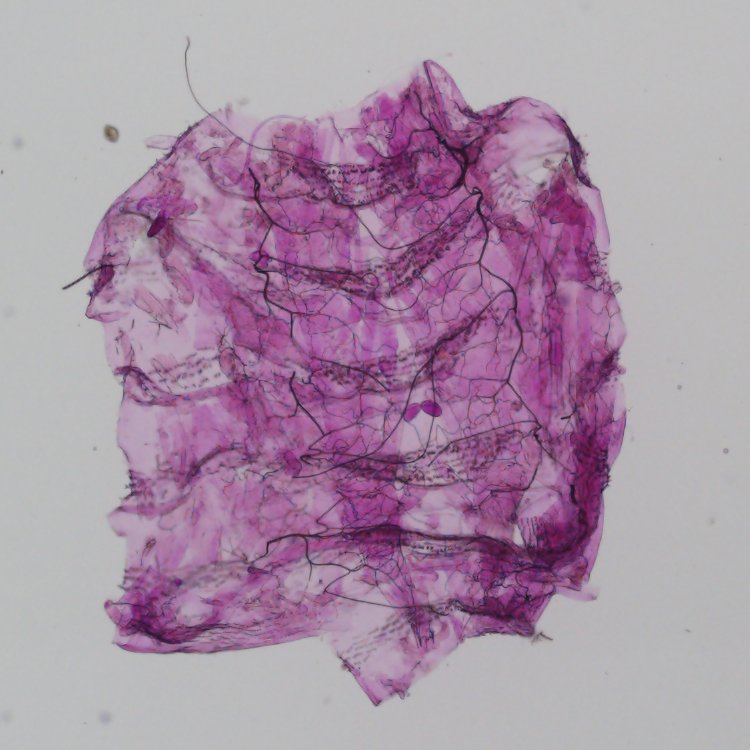

Supplement: Supplementary file 5 — Source Data Fig. 3 [file 44319_2024_102_MOESM5_ESM.zip › Figure 3P.jpg]

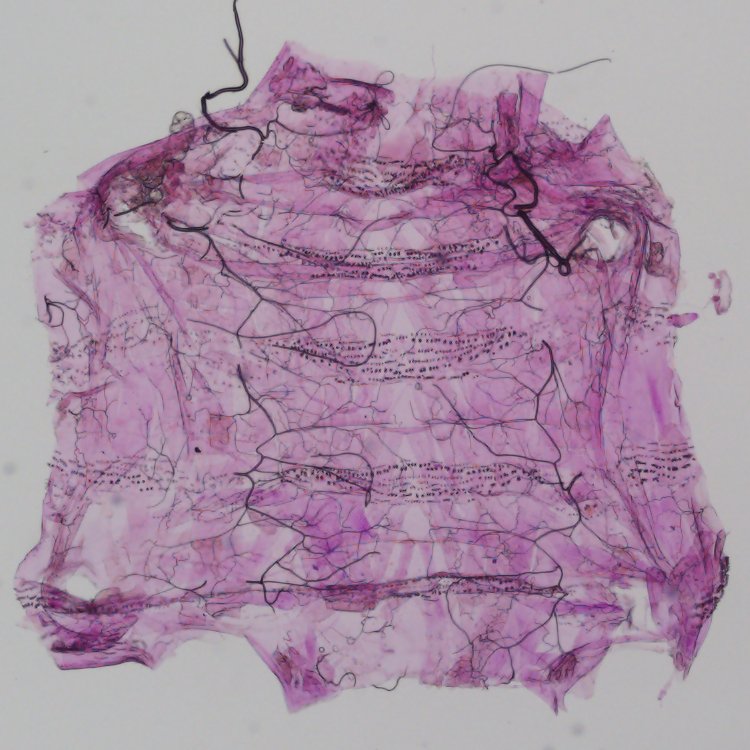

Supplement: Supplementary file 5 — Source Data Fig. 3 [file 44319_2024_102_MOESM5_ESM.zip › Figure 3Q.jpg]

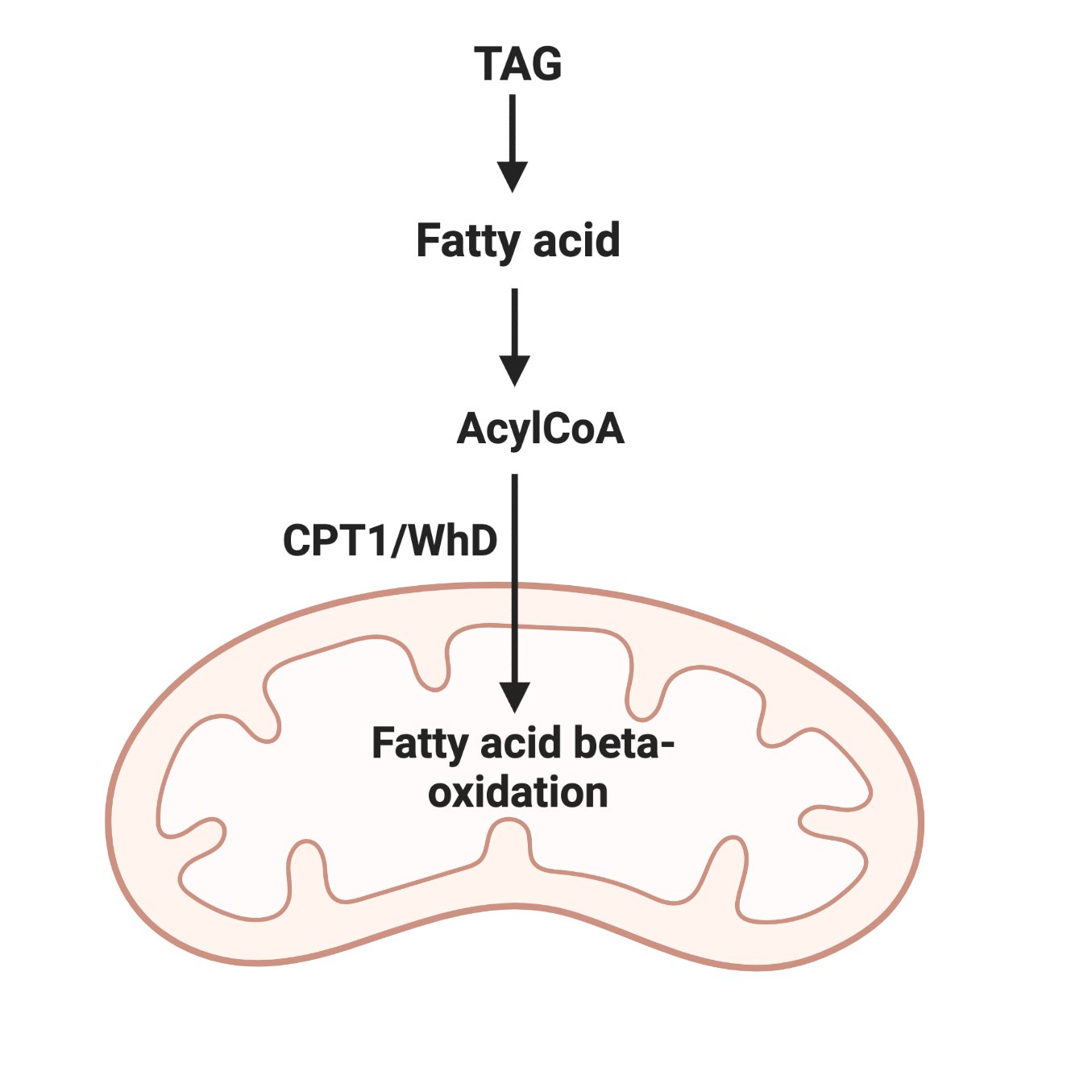

Supplement: Supplementary file 6 — Source Data Fig. 4 [file 44319_2024_102_MOESM6_ESM.zip › Figure 4E.jpg]

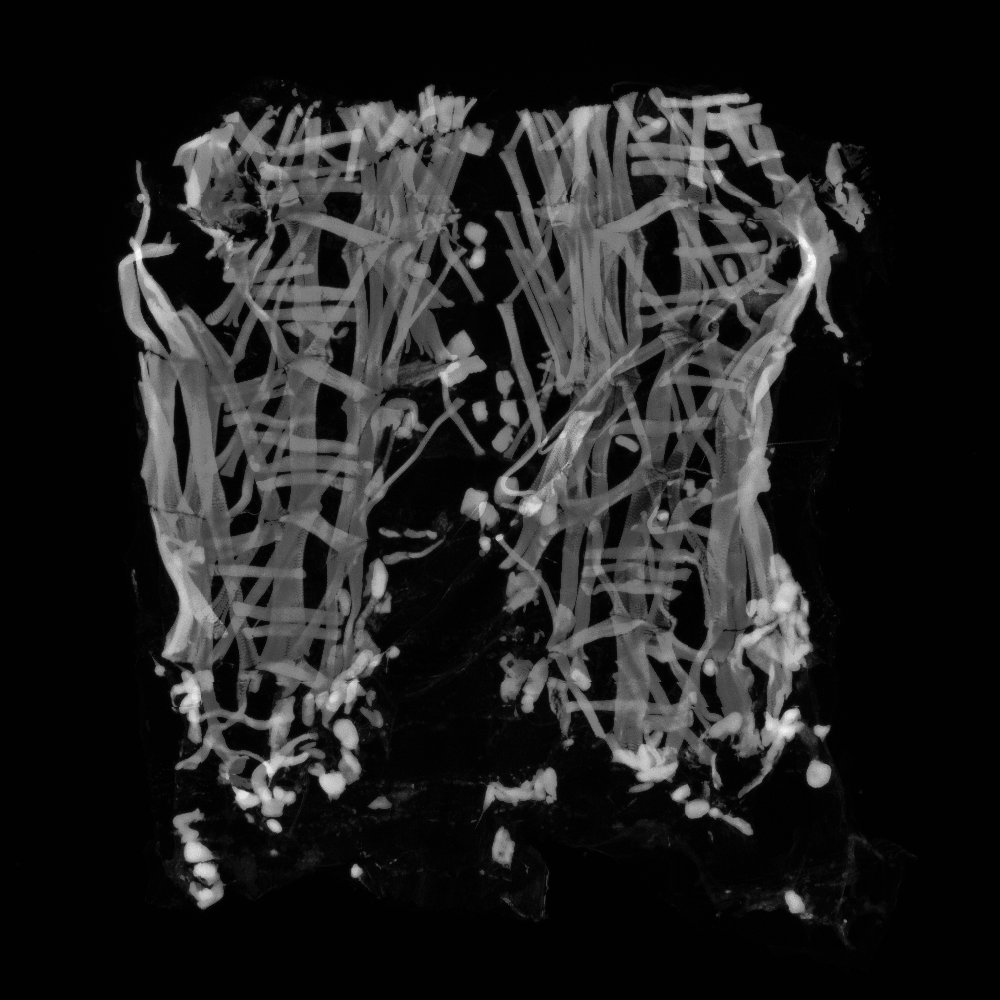

Supplement: Supplementary file 6 — Source Data Fig. 4 [file 44319_2024_102_MOESM6_ESM.zip › Figure 4G.jpg]

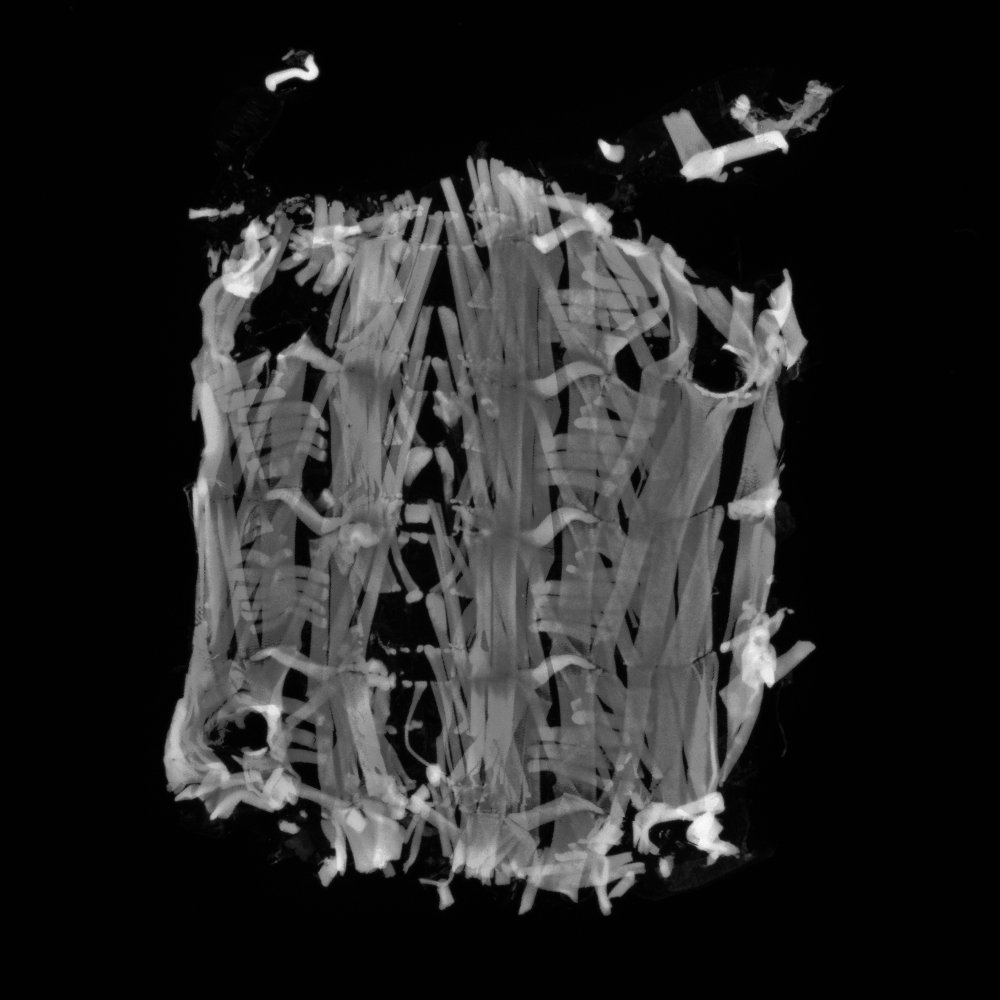

Supplement: Supplementary file 6 — Source Data Fig. 4 [file 44319_2024_102_MOESM6_ESM.zip › Figure 4H.jpg]

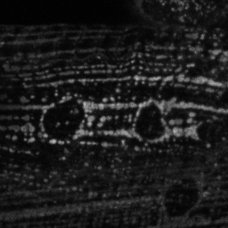

Supplement: Supplementary file 6 — Source Data Fig. 4 [file 44319_2024_102_MOESM6_ESM.zip › Figure 4K.jpg]

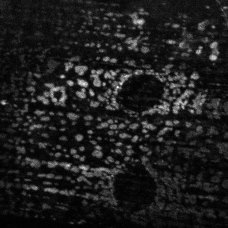

Supplement: Supplementary file 6 — Source Data Fig. 4 [file 44319_2024_102_MOESM6_ESM.zip › Figure 4L.jpg]

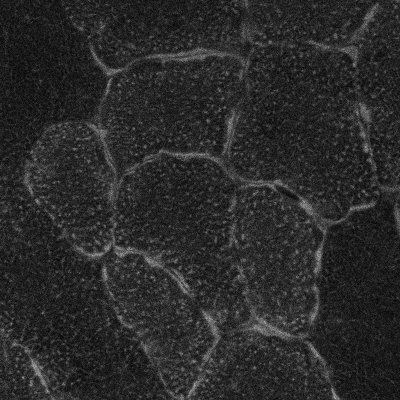

Supplement: Supplementary file 6 — Source Data Fig. 4 [file 44319_2024_102_MOESM6_ESM.zip › Figure 4O'.jpg]

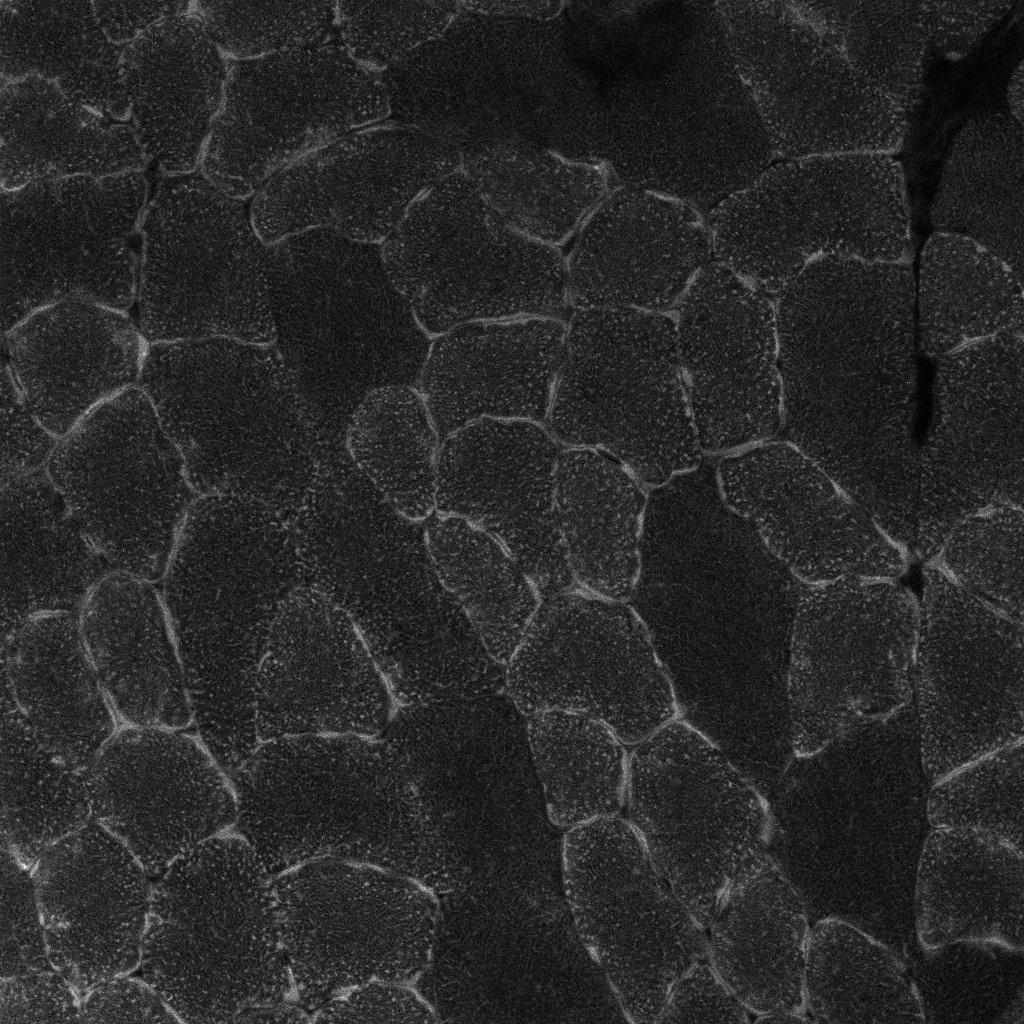

Supplement: Supplementary file 6 — Source Data Fig. 4 [file 44319_2024_102_MOESM6_ESM.zip › Figure 4O.jpg]

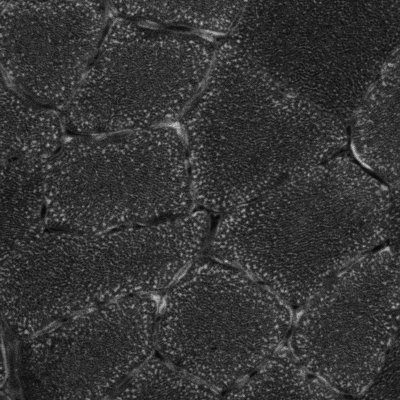

Supplement: Supplementary file 6 — Source Data Fig. 4 [file 44319_2024_102_MOESM6_ESM.zip › Figure 4P'.jpg]

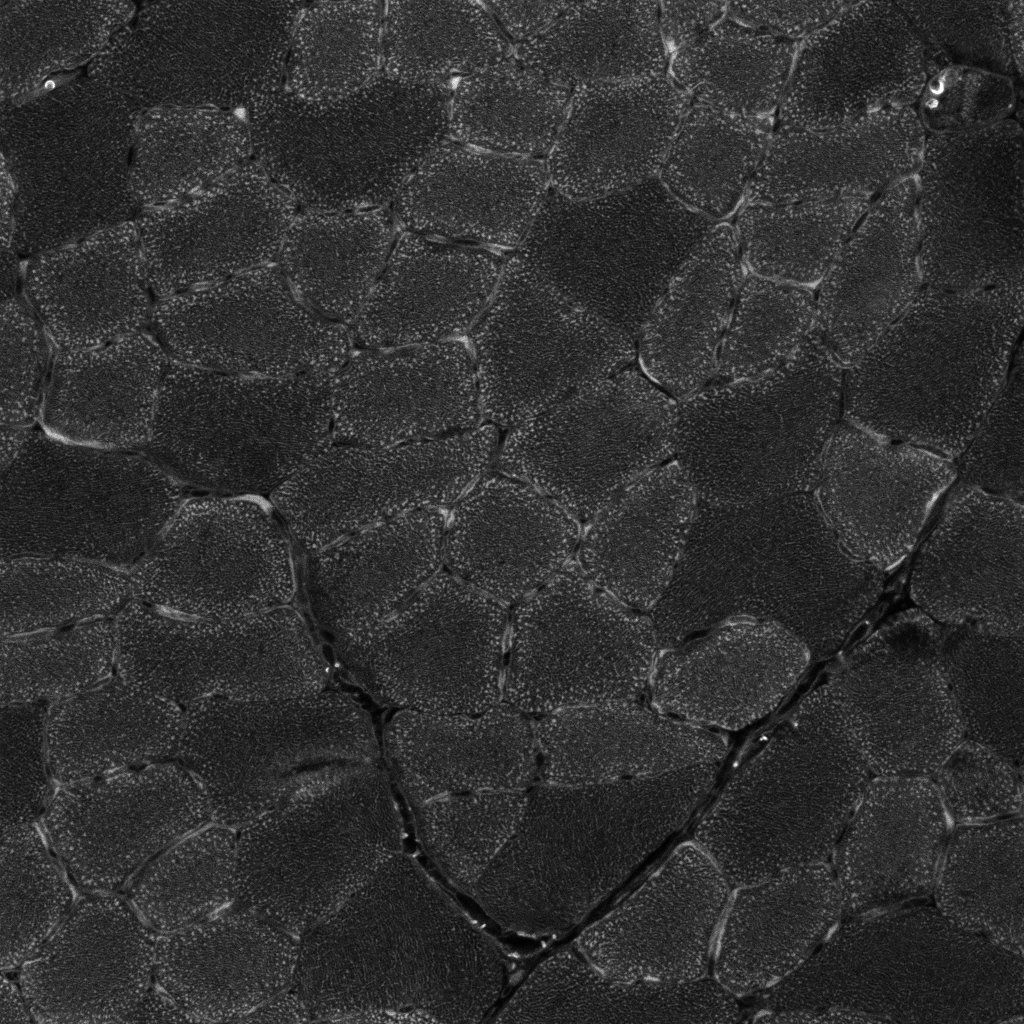

Supplement: Supplementary file 6 — Source Data Fig. 4 [file 44319_2024_102_MOESM6_ESM.zip › Figure 4P.jpg]

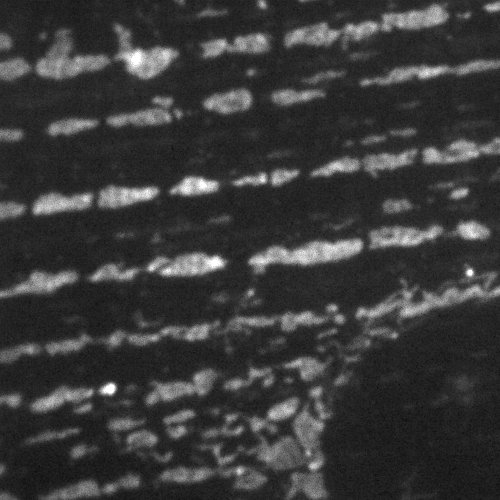

Supplement: Supplementary file 7 — Source Data Fig. 5 [file 44319_2024_102_MOESM7_ESM.zip › Figure 5A.jpg]

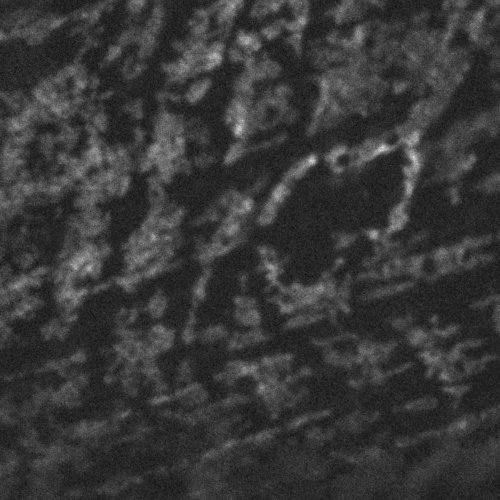

Supplement: Supplementary file 7 — Source Data Fig. 5 [file 44319_2024_102_MOESM7_ESM.zip › Figure 5B.jpg]

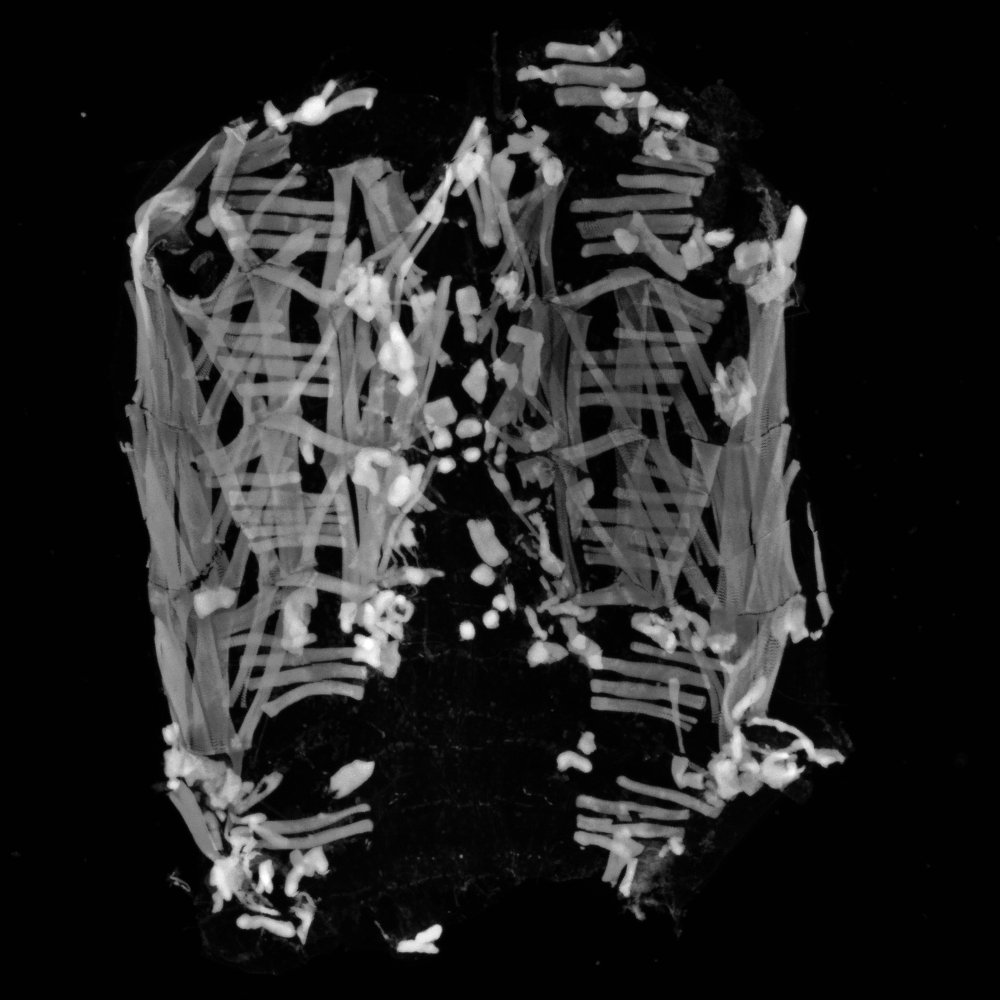

Supplement: Supplementary file 7 — Source Data Fig. 5 [file 44319_2024_102_MOESM7_ESM.zip › Figure 5E.jpg]

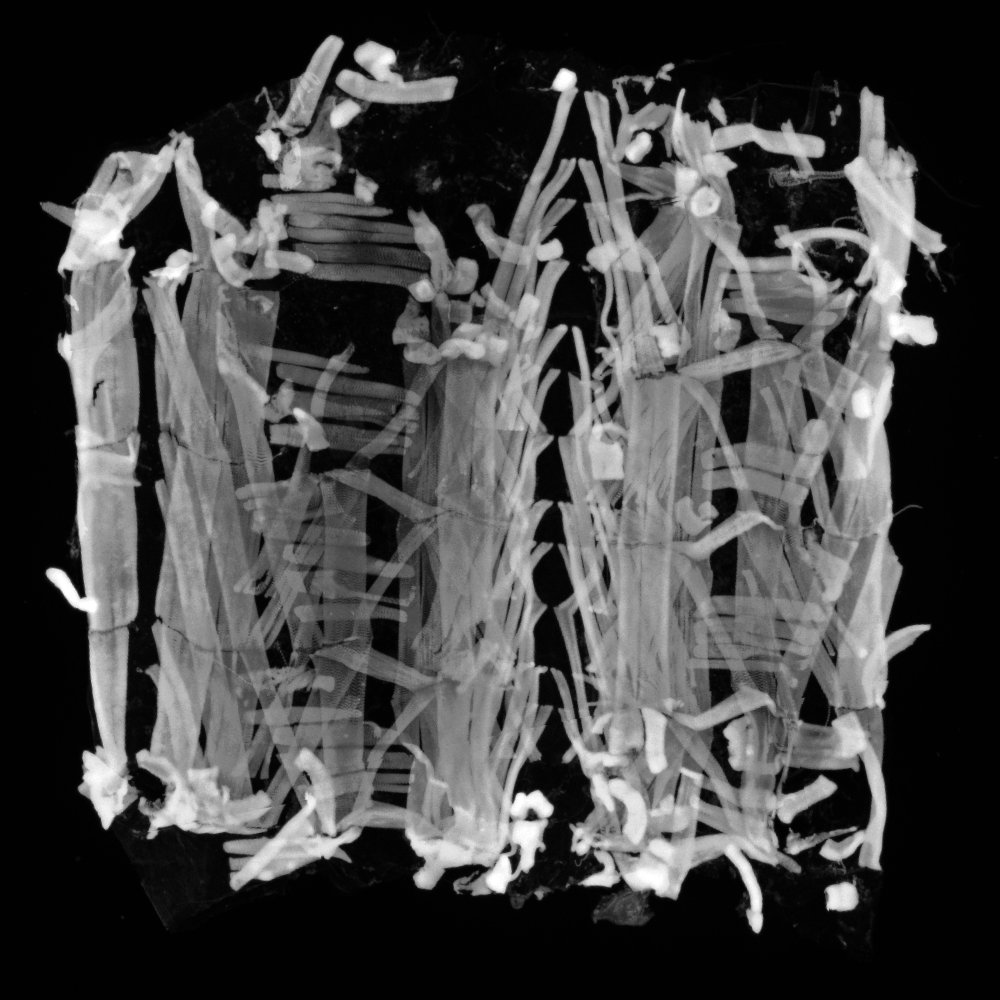

Supplement: Supplementary file 7 — Source Data Fig. 5 [file 44319_2024_102_MOESM7_ESM.zip › Figure 5F.jpg]

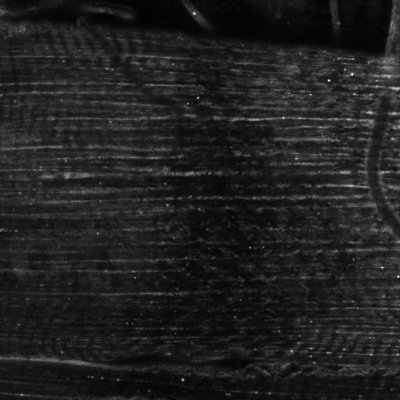

Supplement: Supplementary file 7 — Source Data Fig. 5 [file 44319_2024_102_MOESM7_ESM.zip › Figure 5I.jpg]

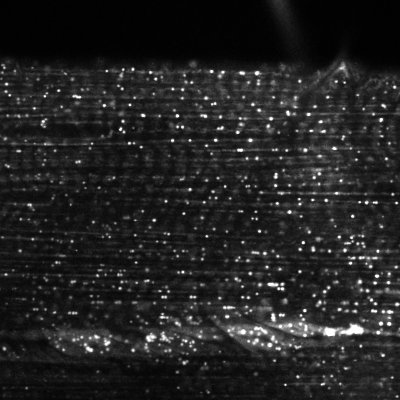

Supplement: Supplementary file 7 — Source Data Fig. 5 [file 44319_2024_102_MOESM7_ESM.zip › Figure 5J.jpg]

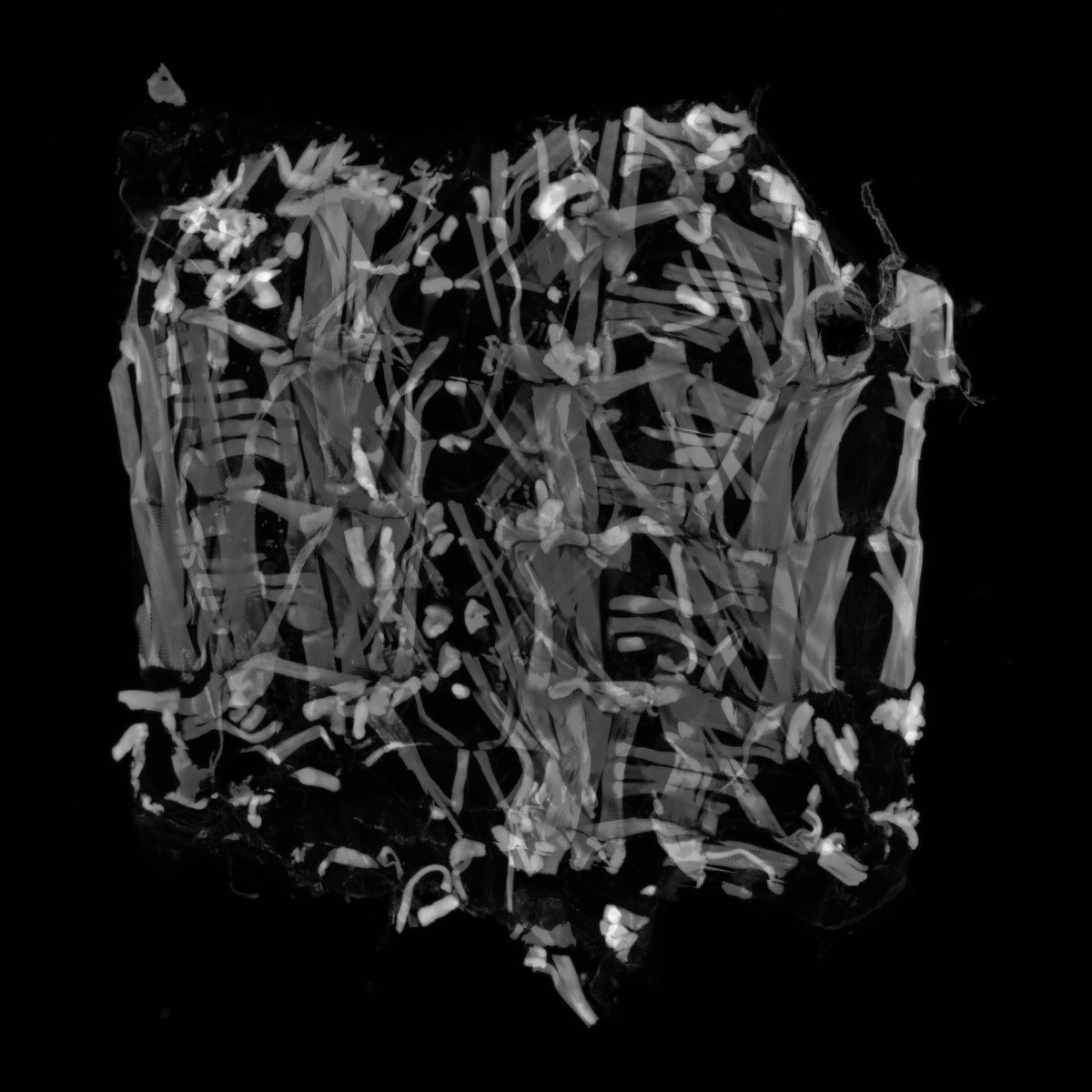

Supplement: Supplementary file 7 — Source Data Fig. 5 [file 44319_2024_102_MOESM7_ESM.zip › Figure 5L.jpg]

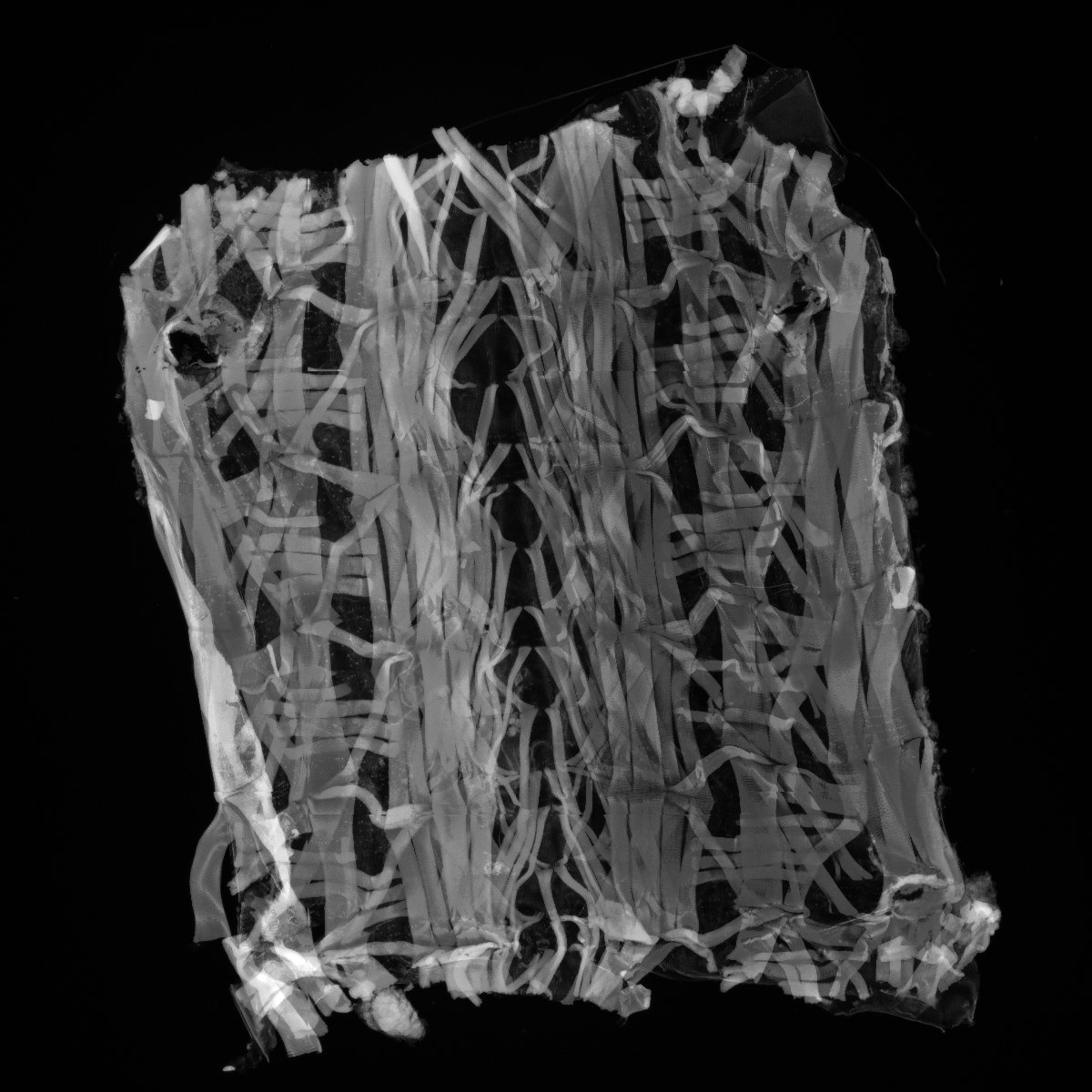

Supplement: Supplementary file 7 — Source Data Fig. 5 [file 44319_2024_102_MOESM7_ESM.zip › Figure 5M.jpg]

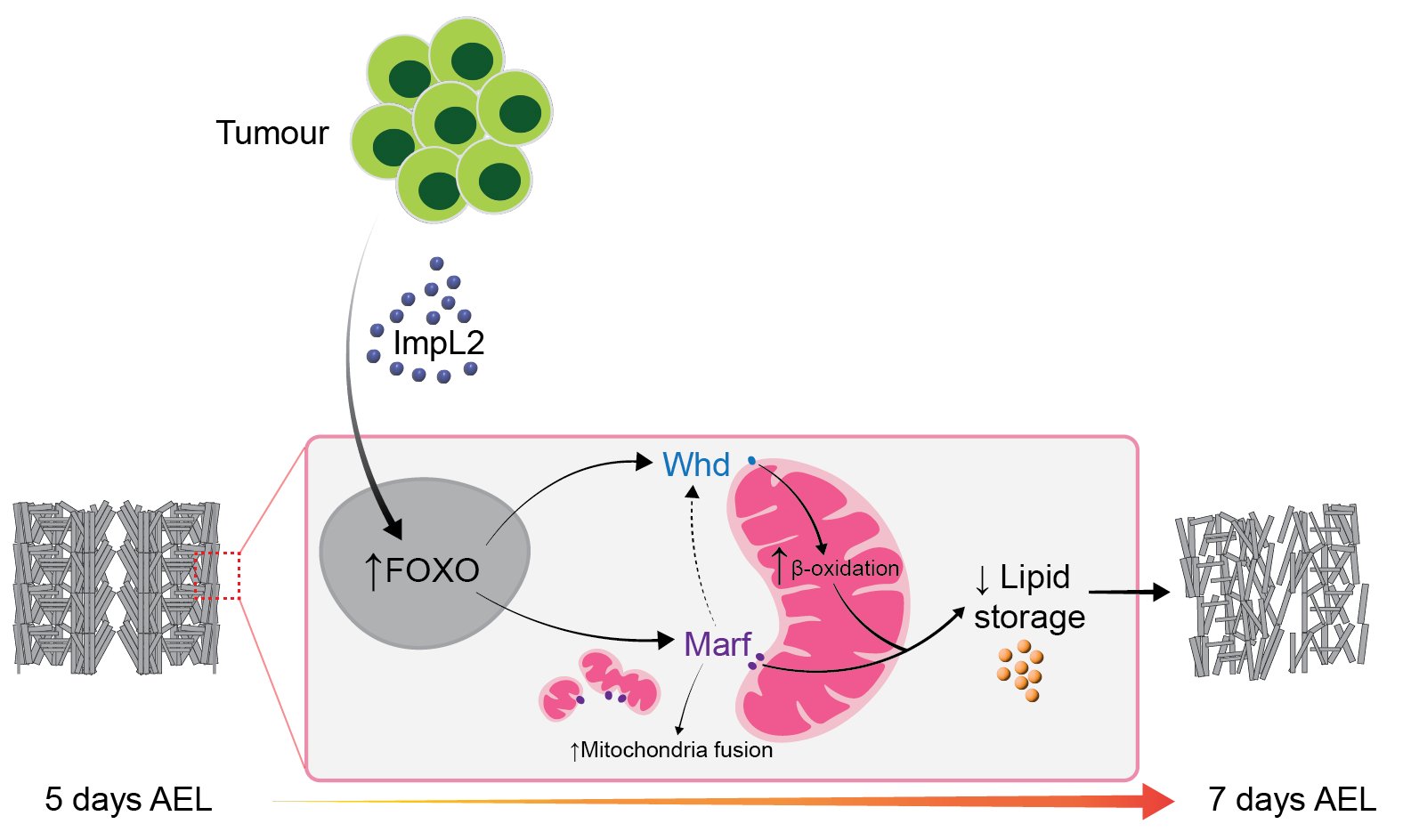

Supplement: Supplementary file 7 — Source Data Fig. 5 [file 44319_2024_102_MOESM7_ESM.zip › Figure 5S.jpg]

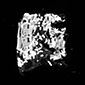

Supplement: Supplementary file 8 — Figure EV Source data [file 44319_2024_102_MOESM8_ESM.zip › EV source data /Figure EV2 Upload Data/Figure EV2H.jpg]

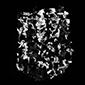

Supplement: Supplementary file 8 — Figure EV Source data [file 44319_2024_102_MOESM8_ESM.zip › EV source data /Figure EV2 Upload Data/Figure EV2E.jpg]

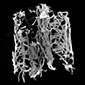

Supplement: Supplementary file 8 — Figure EV Source data [file 44319_2024_102_MOESM8_ESM.zip › EV source data /Figure EV2 Upload Data/Figure EV2D.jpg]

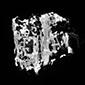

Supplement: Supplementary file 8 — Figure EV Source data [file 44319_2024_102_MOESM8_ESM.zip › EV source data /Figure EV2 Upload Data/Figure EV2G.jpg]

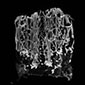

Supplement: Supplementary file 8 — Figure EV Source data [file 44319_2024_102_MOESM8_ESM.zip › EV source data /Figure EV1I-U Upload Data/Figure EV1Q.jpg]

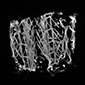

Supplement: Supplementary file 8 — Figure EV Source data [file 44319_2024_102_MOESM8_ESM.zip › EV source data /Figure EV1I-U Upload Data/Figure EV1P.jpg]

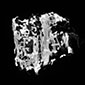

Supplement: Supplementary file 8 — Figure EV Source data [file 44319_2024_102_MOESM8_ESM.zip › EV source data /Figure EV1I-U Upload Data/Figure EV1S.jpg]

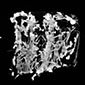

Supplement: Supplementary file 8 — Figure EV Source data [file 44319_2024_102_MOESM8_ESM.zip › EV source data /Figure EV1I-U Upload Data/Figure EV1T.jpg]

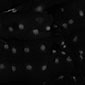

Supplement: Supplementary file 8 — Figure EV Source data [file 44319_2024_102_MOESM8_ESM.zip › EV source data /Figure EV1I-U Upload Data/Figure EV1N.jpg]

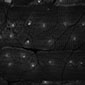

Supplement: Supplementary file 8 — Figure EV Source data [file 44319_2024_102_MOESM8_ESM.zip › EV source data /Figure EV1I-U Upload Data/Figure EV1M.jpg]

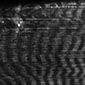

Supplement: Supplementary file 8 — Figure EV Source data [file 44319_2024_102_MOESM8_ESM.zip › EV source data /Figure EV4 Upload Data/Figure EV4A.jpg]

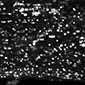

Supplement: Supplementary file 8 — Figure EV Source data [file 44319_2024_102_MOESM8_ESM.zip › EV source data /Figure EV4 Upload Data/Figure EV4B.jpg]

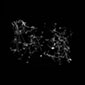

Supplement: Supplementary file 8 — Figure EV Source data [file 44319_2024_102_MOESM8_ESM.zip › EV source data /Figure EV4 Upload Data/Figure EV4E.jpg]

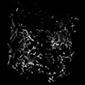

Supplement: Supplementary file 8 — Figure EV Source data [file 44319_2024_102_MOESM8_ESM.zip › EV source data /Figure EV4 Upload Data/Figure EV4D.jpg]

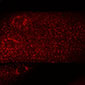

Supplement: Supplementary file 8 — Figure EV Source data [file 44319_2024_102_MOESM8_ESM.zip › EV source data /Figure EV3A-P Upload Data/Figure EV3N.jpg]

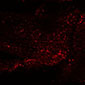

Supplement: Supplementary file 8 — Figure EV Source data [file 44319_2024_102_MOESM8_ESM.zip › EV source data /Figure EV3A-P Upload Data/Figure EV3O.jpg]

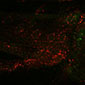

Supplement: Supplementary file 8 — Figure EV Source data [file 44319_2024_102_MOESM8_ESM.zip › EV source data /Figure EV3A-P Upload Data/Figure EV3O''.jpg]

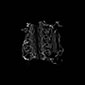

Supplement: Supplementary file 8 — Figure EV Source data [file 44319_2024_102_MOESM8_ESM.zip › EV source data /Figure EV3A-P Upload Data/Figure EV3L.jpg]

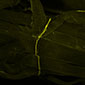

Supplement: Supplementary file 8 — Figure EV Source data [file 44319_2024_102_MOESM8_ESM.zip › EV source data /Figure EV3A-P Upload Data/Figure EV3H.jpg]

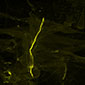

Supplement: Supplementary file 8 — Figure EV Source data [file 44319_2024_102_MOESM8_ESM.zip › EV source data /Figure EV3A-P Upload Data/Figure EV3I.jpg]

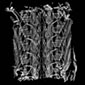

Supplement: Supplementary file 8 — Figure EV Source data [file 44319_2024_102_MOESM8_ESM.zip › EV source data /Figure EV3A-P Upload Data/Figure EV3K.jpg]

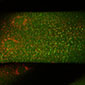

Supplement: Supplementary file 8 — Figure EV Source data [file 44319_2024_102_MOESM8_ESM.zip › EV source data /Figure EV3A-P Upload Data/Figure EV3N''.jpg]

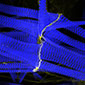

Supplement: Supplementary file 8 — Figure EV Source data [file 44319_2024_102_MOESM8_ESM.zip › EV source data /Figure EV3A-P Upload Data/Figure EV3G'.jpg]

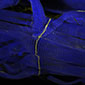

Supplement: Supplementary file 8 — Figure EV Source data [file 44319_2024_102_MOESM8_ESM.zip › EV source data /Figure EV3A-P Upload Data/Figure EV3H'.jpg]

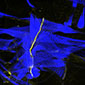

Supplement: Supplementary file 8 — Figure EV Source data [file 44319_2024_102_MOESM8_ESM.zip › EV source data /Figure EV3A-P Upload Data/Figure EV3I'.jpg]

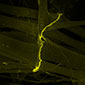

Supplement: Supplementary file 8 — Figure EV Source data [file 44319_2024_102_MOESM8_ESM.zip › EV source data /Figure EV3A-P Upload Data/Figure EV3G.jpg]

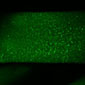

Supplement: Supplementary file 8 — Figure EV Source data [file 44319_2024_102_MOESM8_ESM.zip › EV source data /Figure EV3A-P Upload Data/Figure EV3N'.jpg]

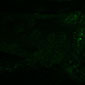

Supplement: Supplementary file 8 — Figure EV Source data [file 44319_2024_102_MOESM8_ESM.zip › EV source data /Figure EV3A-P Upload Data/Figure EV3O'.jpg]
