# Supplementary material for: Mitochondrial fusion and altered beta-oxidation drive muscle wasting in a Drosophila cachexia model
Source: EMBO Rep. 2024 Mar 1;25(4):15. doi: 10.1038/s44319-024-00102-z (PMC11014992; doi:10.1038/s44319-024-00102-z)
Supplement: Supplementary file 10 — Expanded View Figures [file 44319_2024_102_MOESM10_ESM.pdf]

Expanded View Figures

Figure EV1. Mitochondria size/membrane potential in *QRas^{V12}scrib^{RNAi}*, manipulation of developmental delay does not alter mitochondria size or membrane potential and ROS manipulations in the muscles of tumour-bearing animals.

(**A**) Size distribution of individual mitochondria in the muscles of control (5 AEL) and *Ras^{V12}dlg1^{RNAi}* larvae (4–7 AEL) ($n = 1351, 1950, 1313, 1285, 1214$). (**B**) Proportion of small, medium, and large mitochondria as a percentage of total mitochondria in control (5 AEL) and *QRas^{V12}scrib^{RNAi};MhcGal4* muscles (4–7 AEL), performed using Chi-square test ($P = 0.72$, $n = 3, 3, 3, 3, 3$). (**C**) Size distribution of individual mitochondria in the muscles of control (5 AEL) and *QRas^{V12}scrib^{RNAi};MhcGal4* muscles (4–7 AEL) ($n = 427, 609, 841, 589, 717$). (**D**–**E**″) Zoomed in images of control (5 AEL) and *Ras^{V12}dlg1^{RNAi}* (7 AEL) larval muscles stained with MitoTracker™ Green which labels all mitochondria, and active mitochondria with TMRE (zoomed out images are in Fig. 1J–K″). White arrows indicate an example of a mitochondrion that has no TMRE membrane potential. (**F**-**G**″) Representative images of control (5 days AEL) and *QRas^{V12}scrib^{RNAi};MhcGAL4* (6 days AEL) larval muscle fillets stained with MitoTracker™ Green which labels all mitochondria (**F**, **G**), and active mitochondria with TMRE (**F′**, **G′**). (**H**) Quantification of the percentage of total mitochondria stained with MitoTracker™ Green that are also positive for TMRE in control (5 AEL) and *QRas^{V12}scrib^{RNAi};MhcGAL4* (6 AEL) performed using Student's *t* test ($n = 5, 5$). (**I**) Quantification of normalised muscle ATP measured in control (5 AEL) and *QRas^{V12}scrib^{RNAi};MhcGAL4* (6 AEL), performed using Student's *t* test ($n = 3, 3$). (**J**) Proportion of small, medium, and large mitochondria as a percentage of total mitochondria in control (5 AEL) and *phmGAL4>torso^{RNAi}* (7 AEL) animals, performed using Chi-square test ($P > 0.99$, $n = 4, 5$). (**K**) Size distribution of individual mitochondria in the muscles of control (5 AEL) and *phmGAL4>torso^{RNAi}* (7 AEL) animals ($n = 2815, 2959$). (**L**) Quantification of the percentage of total mitochondria stained with MitoTracker™ Green that are shown to be active via TMRE incorporation in the muscles of control (5 AEL) and *phmGAL4>torso^{RNAi}* (7 AEL), performed using Student's *t* test ($n = 4, 4$). (**M**, **N**) Representative images of DHE staining in the muscles of control (6 AEL) and *QRas^{V12}scrib^{RNAi};Mef2GAL4 > UAS Luciferase* (7 AEL) larvae. (**O**) Quantification of DHE staining in M-N, performed using Mann–Whitney *U* ($n = 45, 15$).
(**P**, **Q**) Representative muscle fillets from *QRas^{V12}scrib^{RNAi};Mef2GAL4>lacz^{RNAi};mCherry^{RNAi}* and *QRas^{V12}scrib^{RNAi};Mef2GAL4 > UAS CatalaseA;UAS Sod1* larvae (both 7 AEL), stained with Phalloidin to visualise actin. (**R**) Quantification of muscle integrity in (**P**, **Q**) performed using Student's *t* test ($n = 13, 11$). (**S**, **T**) Representative muscle fillets from *QRas^{V12}scrib^{RNAi};MhcGAL4>mCherry^{RNAi}*, *QRas^{V12}scrib^{RNAi};MhcGAL4 > UAS GPx1* (7 days AEL) stained with Phalloidin to visualise actin. This data was part of an experiment with EV2 G-H and EV3 FF-GG, which use the same controls. (**U**) Quantification of muscle integrity in (**S**, **T**), performed using Kruskal-Wallis as part of an analysis with EV2 I and EV3 HH, which use the same controls ($n = 13, 16$). Scale bars: 10 μm for (**D**, **D′**, **D″**, **E**, **E′**, **E″**), 20 μm for (**M**, **N**), and 500 μm for (**F**, **F′**, **F″**, **G**, **G′**, **G″**, **P**, **Q**, **S**, **T**). Data information: All error bars are +/− SEM. *P* values are: ns (not significant), $P < 0.05$; $^{**}P < 0.001$; $^{****}P < 0.0001$. Source data are available online for this figure.

 © The Author(s)

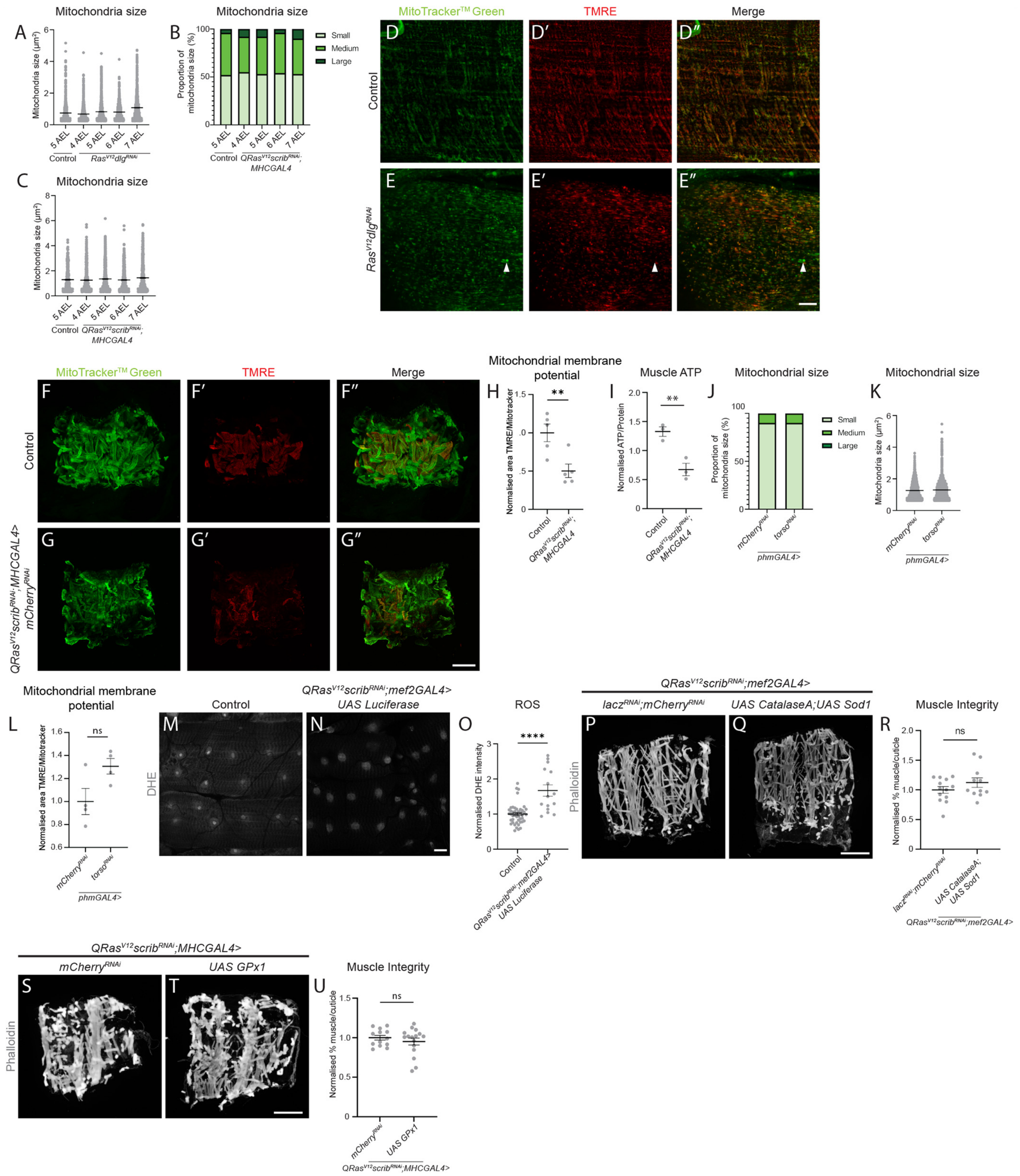
A
Mitochondria size
Mitochondria size (μm²)
5 AEL
4 AEL
5 AEL
6 AEL
7 AEL
Control
Ras^{V12}dlg^{RNAi}
B
Mitochondria size
Proportion of mitochondria size (%)
Small
Medium
Large
Control
QRas^{V12}scrib^{RNAi};
MHCGAL4
C
Mitochondria size
Mitochondria size (μm²)
D
D′
D″
MitoTracker™ Green
TMRE
Merge
Control
E
E′
E″
Ras^{V12}dlg^{RNAi}
F
F′
F″
G
G′
G″
QRas^{V12}scrib^{RNAi};MHCGAL4>
mCherry^{RNAi}
H
Mitochondrial membrane potential
Normalised area TMRE/Mitotracker
**
I
Muscle ATP
Normalised ATP/Protein
**
J
Mitochondrial size
Proportion of mitochondria size (%)
mCherry^{RNAi}
torso^{RNAi}
phmGAL4>
K
Mitochondrial size
Mitochondria size (μm²)
L
Mitochondrial membrane potential
Normalised area TMRE/Mitotracker
ns
M
N
DHE
Control
QRas^{V12}scrib^{RNAi};mef2GAL4>
UAS Luciferase
O
ROS
Normalised DHE intensity
****
QRas^{V12}scrib^{RNAi};mef2GAL4>
UAS Luciferase
P
Q
lacz^{RNAi};mCherry^{RNAi}
UAS CatalaseA;UAS Sod1
Phalloidin
R
Muscle Integrity
Normalised % muscle/cuticle
ns
S
T
QRas^{V12}scrib^{RNAi};MHCGAL4>
UAS GPx1
U
Muscle Integrity
ns

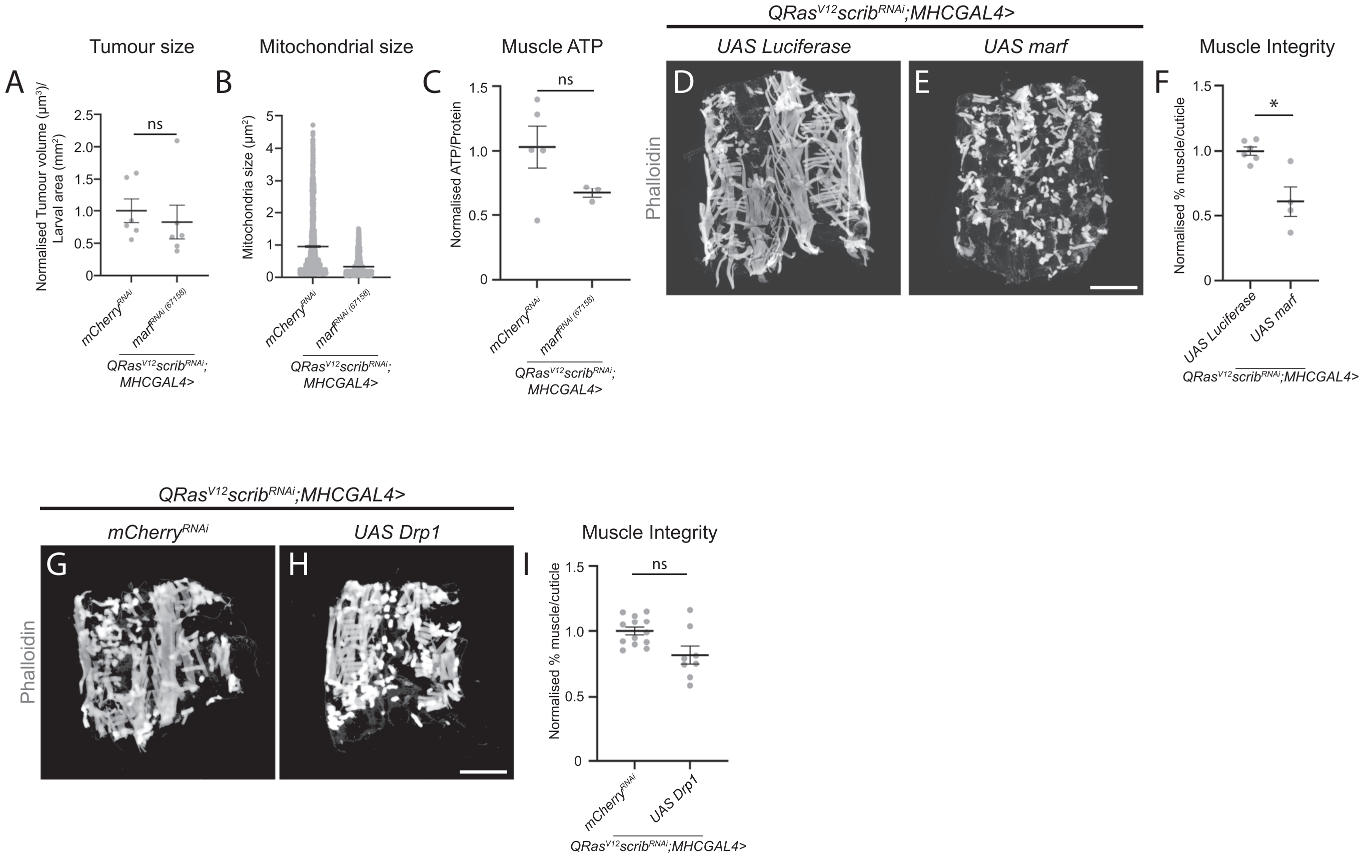

Figure EV2. Muscle mitochondria size, integrity, ATP tumour animals with muscle-specific marf and Drp1 overexpression compared to control.

(**A**) Quantification of normalised tumour volume of *$QRas^{V12}scrib^{RNAi}$;MhcGAL4>$mCherry^{RNAi}$* and *$QRas^{V12}scrib^{RNAi}$;MhcGAL4>$marf^{RNAi\ (67158)}$* larvae (7 AEL), performed using Kruskal–Wallis as part of an analysis with EV3 F and EV5 A, which use the same controls ($n = 6, 6$). (**B**) Size distribution of individual mitochondria in the muscles of *$QRas^{V12}scrib^{RNAi}$;MhcGAL4>$mCherry^{RNAi}$* and *$QRas^{V12}scrib^{RNAi}$;MhcGAL4>$marf^{RNAi\ (67158)}$* larvae (6 AEL) ($n = 1516, 1690$). (**C**) Quantification of normalised muscle ATP in *$QRas^{V12}scrib^{RNAi}$;MhcGAL4>$mCherry^{RNAi}$* and *$QRas^{V12}scrib^{RNAi}$;MhcGAL4>$marf^{RNAi\ (67158)}$* larvae (6 AEL), performed using one-way ANOVA as part of an analysis with EV3 E and EV5 B, which use the same controls ($n = 5, 3$). (**D**, **E**) Representative images of muscle fillets of *$QRas^{V12}scrib^{RNAi}$;MhcGAL4 > UAS Luciferase* and *$QRas^{V12}scrib^{RNAi}$;MhcGAL4 > UAS-marf* larvae (7 AEL). (**F**) Quantification of muscle integrity of *$QRas^{V12}scrib^{RNAi}$;MhcGAL4 > UAS Luciferase* and *$QRas^{V12}scrib^{RNAi}$;MhcGAL4 > UAS-marf* larvae (7 AEL), performed using Welch's *t* test ($n = 6, 4$). (**G**, **H**) Representative images of muscle fillets of *$QRas^{V12}scrib^{RNAi}$;MhcGAL4>$mCherry^{RNAi}$* and *$QRas^{V12}scrib^{RNAi}$;MhcGAL4 > UAS Drp1* (7 AEL). This data was part of an experiment with EV1 S–T and EV3 FF–GG, which use the same controls. (**I**) Quantification of muscle integrity of *$QRas^{V12}scrib^{RNAi}$;MhcGAL4>$mCherry^{RNAi}$* and *$QRas^{V12}scrib^{RNAi}$;MhcGAL4 > UAS Drp1* (7 AEL), performed using Kruskal–Wallis as part of an analysis with EV1 U and EV3 HH, which use the same controls ($n = 13, 8$). Scale bars: 500 µm for (**D**, **E**, **G**, **H**). Data information: All error bars are +/− SEM. *P* values are: ns (not significant), $^{*}P < 0.05$. Source data are available online for this figure.

 © The Author(s)

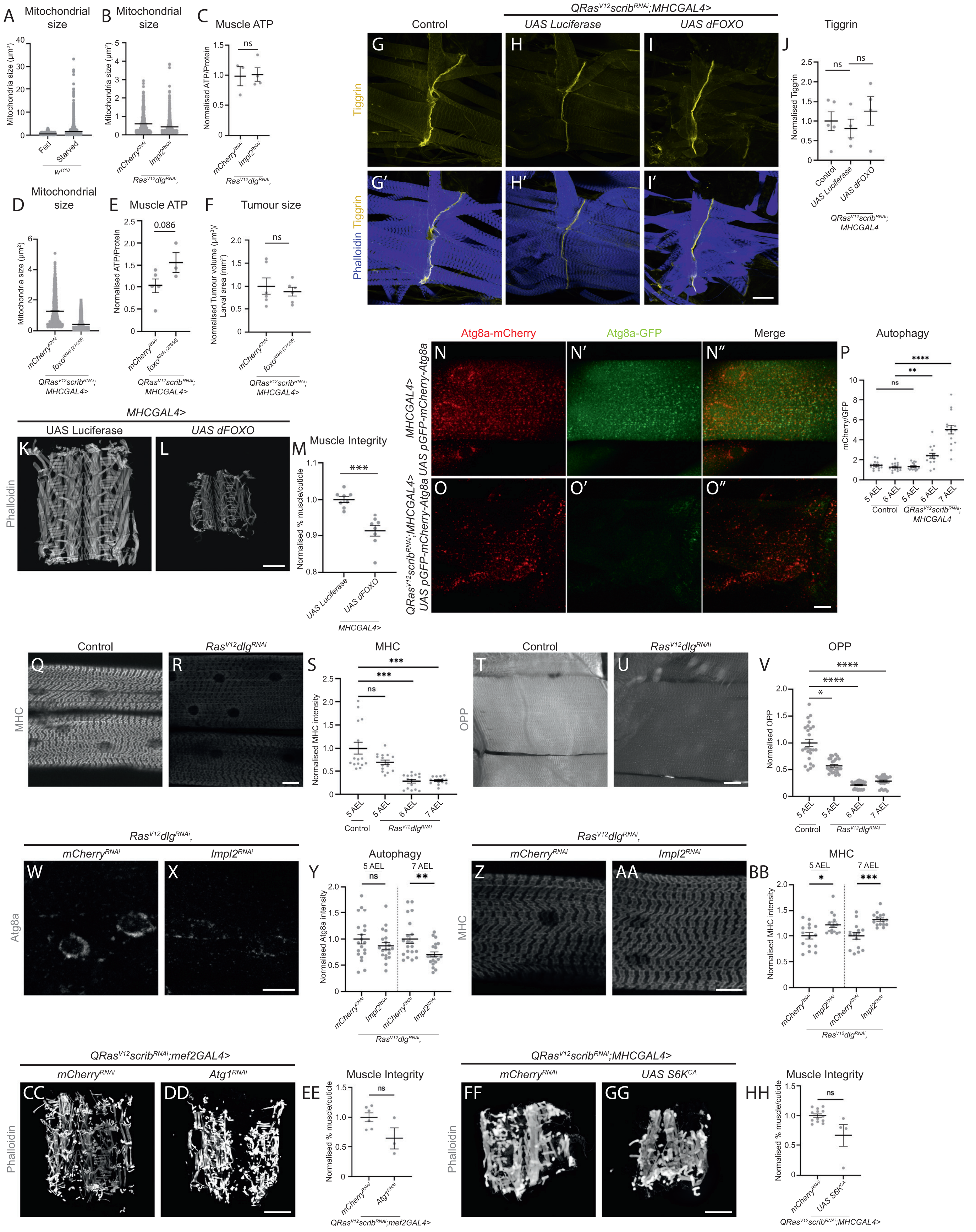
A Mitochondrial size
B Mitochondrial size
C Muscle ATP
Mitochondria size (µm²)
Fed
Starved
w^{1118}
mCherry^{RNAi}
Impl2^{RNAi}
Ras^{V12}dlg^{RNAi},
Normalised ATP/Protein
ns
D Mitochondrial size
E Muscle ATP
F Tumour size
0.086
foxo$^{RNAi (27656)}$
QRas^{V12}scrib^{RNAi}; MHCGAL4>
Normalised Tumour volume (µm³)/ Larval area (mm²)
QRas^{V12}scrib^{RNAi};MHCGAL4>
Control
UAS Luciferase
UAS dFOXO
Tiggrin
Phalloidin Tiggrin
J Tiggrin
Normalised Tiggrin
MHCGAL4>
Phalloidin
M Muscle Integrity
Normalised % muscle/cuticle
***
Atg8a-mCherry
Atg8a-GFP
Merge
MHCGAL4> UAS pGFP-mCherry-Atg8a
QRas^{V12}scrib^{RNAi};MHCGAL4> UAS pGFP-mCherry-Atg8a
P Autophagy
mCherry/GFP
****
**
5 AEL
6 AEL
7 AEL
QRas^{V12}scrib^{RNAi}; MHCGAL4
Ras^{V12}dlg^{RNAi}
MHC
S MHC
Normalised MHC intensity
OPP
V OPP
Normalised OPP
*
****
Atg8a
Y Autophagy
Normalised Atg8a intensity
BB MHC
QRas^{V12}scrib^{RNAi};mef2GAL4>
Atg1^{RNAi}
EE Muscle Integrity
UAS S6K^{CA}
HH Muscle Integrity

Figure EV3. Assessment of mitochondrial size, muscle ATP, tumour size, autophagy and translation under various genetic manipulations.

(**A**) Size distribution of individual mitochondria in the muscles of w^{1118} larvae raised on a normal diet (Fed) and w^{1118} larvae undergoing nutrient restriction (Starved) from critical weight (60 h after larval hatching (ALH)) (5 AEL, $n = 2248, 3747$). (**B**) Size distribution of individual mitochondria in the muscles of *Ras^{V12}dlg1^{RNAi},mCherry^{RNAi}* and *Ras^{V12}dlg1^{RNAi},ImpL2^{RNAi}* larvae (7 AEL, $n = 432, 1037$). (**C**) Quantification of normalised muscle ATP of 7 AEL *Ras^{V12}dlg1^{RNAi},mCherry^{RNAi}* and *Ras^{V12}dlg1^{RNAi},ImpL2^{RNAi}* larvae, performed using Student's *t* test ($n = 3, 4$). (**D**) Size distribution of individual mitochondria in the muscles of *QRas^{V12}scrib^{RNAi};MhcGAL4>mCherry^{RNAi}*, and *QRas^{V12}scrib^{RNAi};MhcGAL4>foxo$^{RNAi\ (27656)}$* larvae (6 AEL, n = 1083, 2230). (**E**) Quantification of normalised muscle ATP of 6 AEL *QRas^{V12}scrib^{RNAi};MhcGAL4>mCherry^{RNAi}*, and *QRas^{V12}scrib^{RNAi};MhcGAL4>foxo$^{RNAi\ (27656)}$* larvae, performed using one-way ANOVA as part of an analysis with EV2 C and EV5 B, which use the same controls ($n = 5, 3$). (**F**) Quantification of normalised tumour volume of 7 AEL *QRas^{V12}scrib^{RNAi};MhcGAL4>mCherry^{RNAi}*, and *QRas^{V12}scrib^{RNAi};MhcGAL4>foxo$^{RNAi\ (27656)}$* larvae, performed using Kruskal–Wallis as part of an analysis with EV2 A and EV5 A, which use the same controls ($n = 6, 6$). (**G–I′**) Representative images of Tiggrin staining marking a muscle/tendon junction in control (5 AEL, **G**, **G′**), *QRas^{V12}scrib^{RNAi};MhcGAL4 > UAS Luciferase* (5 AEL, **H**, **H′**) and *QRas^{V12}scrib^{RNAi};MhcGAL4 > UAS dFOXO* (5 AEL, **I**, **I′**) larval muscle. (**J**) Quantification of Tiggrin levels in control (5 AEL), *QRas^{V12}scrib^{RNAi};MhcGAL4 > UAS Luciferase* and *QRas^{V12}scrib^{RNAi};MhcGAL4 > UAS dFOXO* (5 AEL) larval muscles, performed using one-way ANOVA ($n = 5, 4, 4$). (**K**, **L**) Representative images of muscle fillets from *MhcGAL4 > UAS Luciferase*, *MhcGAL4 > UAS dFOXO* (both 5 AEL). (**M**) Quantification of muscle integrity of *MhcGAL4 > UAS Luciferase* and *MhcGAL4 > UAS dFOXO* (5 AEL), performed using Student's *t* test, ($n = 8, 8$). (**N–O″**) Representative images of larval muscles of *MhcGAL4* (5 AEL).and *QRas^{V12}scrib^{RNAi};MhcGAL4* animals (7 AEL) crossed to a reporter of autophagy, Atg8a, tagged with both mCherry (**N**, **O**) and GFP (**N′**,**O′**). Merged images are shown in (**N″**, **O″**). (**P**) Quantification of the ratio of Atg8a-mCherry to Atg8a-GFP in control and *Ras^{V12}dlg1^{RNAi}* larvae at days 5–7 AEL, performed using Brown–Forsythe ($n = 15, 15, 15, 15, 15$). (**Q**, **R**) Representative images of Myosin Heavy chain (Mhc) staining in the muscles of control (5 AEL) and *Ras^{V12}dlg1^{RNAi}* (7 AEL) larvae. (**S**) Quantification of Mhc staining in control and *Ras^{V12}dlg1^{RNAi}* larvae from days 5–7 AEL, performed using Brown–Forsythe ($n = 15, 15, 15, 15$). (**T**, **U**) Representative images of OPP staining in the muscles of control (5 AEL) and *Ras^{V12}dlg1^{RNAi}* (7 AEL) larvae. (**V**) Quantification of OPP staining in the muscles of control (5 AEL) and *Ras^{V12}dlg1^{RNAi}* (7 AEL) larvae, performed using Kruskal–Wallis ($n = 25, 25, 25, 25$). (**W**, **X**) Representative images of muscles of *Ras^{V12}dlg1^{RNAi},mCherry^{RNAi}* (7 AEL) and *Ras^{V12}dlg1^{RNAi},ImpL2^{RNAi}* (7 AEL) larvae crossed to a reporter of autophagy, Atg8a, tagged with mCherry. (**Y**) Quantification of Atg8a-mCherry levels in (**W**, **X**), as well as from earlier timepoints, performed using Student's *t* test (5 days AEL), Welch's *t* test (6 days AEL), and Mann–Whitney *U* (7 days AEL, $n = 20, 20, 20, 20, 20, 20$). (**Z**, **AA**) Representative images of Myosin Heavy chain (Mhc) staining in the muscles of *Ras^{V12}dlg1^{RNAi},mCherry^{RNAi}* (7 AEL) and *Ras^{V12}dlg1^{RNAi},ImpL2^{RNAi}* (7 AEL) larvae. (**BB**) Quantification of Mhc staining in (**Z**, **AA**), as well as staining from earlier timepoints, performed using Mann–Whitney U (5 and 6 days AEL) and Welch's *t* test (7 days AEL, $n = 15, 15, 15, 15, 15, 15$). (**CC**, **DD**) Representative muscle fillets from *QRas^{V12}scrib^{RNAi};Mef2GAL4>mCherry^{RNAi}* and *QRas^{V12}scrib^{RNAi};Mef2GAL4>Atg1^{RNAi}* larvae (both 7 AEL), stained with Phalloidin to visualise actin. (**EE**) Quantification of muscle integrity in (**CC**, **DD**) performed using Student's *t* test ($n = 6, 3$).
(**FF**, **GG**) Representative muscle fillets from *QRas^{V12}scrib^{RNAi};MhcGAL4>mCherry^{RNAi}*, *QRas^{V12}scrib^{RNAi};MhcGAL4 > UAS-S6K^{CA}* larvae (both 7 AEL), stained with Phalloidin to visualise actin. This data was part of an experiment with EV1 S–T and EV2 G-H, which use the same controls. (**HH**) Quantification of muscle integrity in (**FF**, **GG**) performed using Kruskal–Wallis as part of an analysis with EV1 U and EV2 I, which used the same controls ($n = 13, 4$). Scale bars: 20 µm for (**N**, **N′**, **N″**, **O**, **O′**, **O″**, **Q**, **R**, **T**, **U**, **W**, **X**, **Z**, **AA**), 50 µm for (**G**, **G′**, **H**, **H′**, **I**, **I′**), and 500 µm for (**K**, **L**, **CC**, **DD**, **FF**, **GG**). Data information: All error bars are +/− SEM. *P* values are: ns (not significant), $P > 0.05$; $^*P < 0.05$; $^{**}P < 0.01$; $^{***}P < 0.001$; $^{****}P < 0.0001$. Source data are available online for this figure.

 © The Author(s)

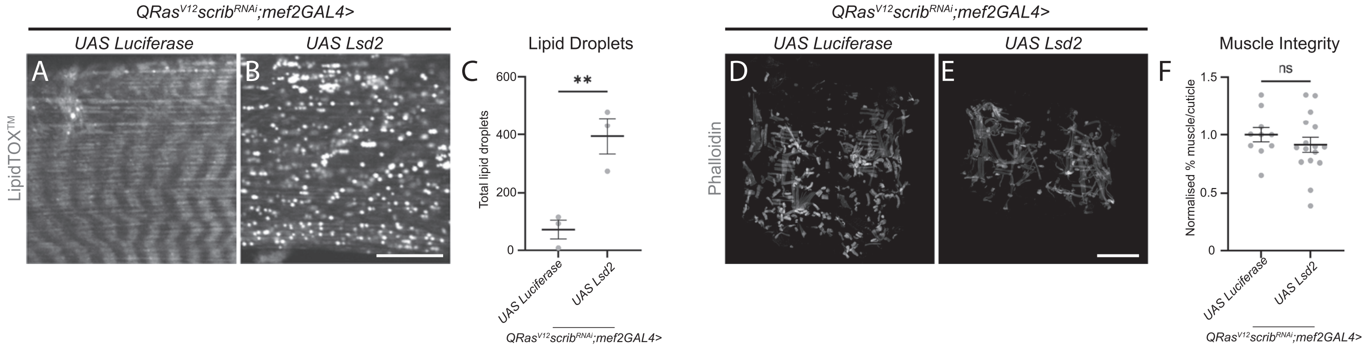

Figure EV4. Increasing storage of lipids in lipid droplets does not rescue muscle integrity.

(**A**, **B**) Lipid droplets stained with LipidTOX™ in the muscles of *QRas^{V12}scrib^{RNAi};Mef2GAL4 > UAS Luciferase* and *QRas^{V12}scrib^{RNAi};Mef2GAL4 > UAS Lsd2* larvae (6 AEL). (**C**) Quantification of the number of LDs/mm^2 in (**A**, **B**) performed using Student's t test ($n = 3, 3$). (**D**, **E**) Representative muscle fillets from *QRas^{V12}scrib^{RNAi};Mef2GAL4 > UAS Luciferase* and *QRas^{V12}scrib^{RNAi};Mef2GAL4 > UAS Lsd2* larvae (7 AEL), stained with Phalloidin to visualise actin. (**F**) Quantification of muscle integrity in N and O, performed using Student's t test ($n = 10, 16$). Scale bars: 20 µm for (**A**, **B**), and 500 µm for (**D**, **E**). Data information: All error bars are +/− SEM. P values are: ns (not significant), $P > 0.05$; $^{**}P < 0.01$. Source data are available online for this figure.

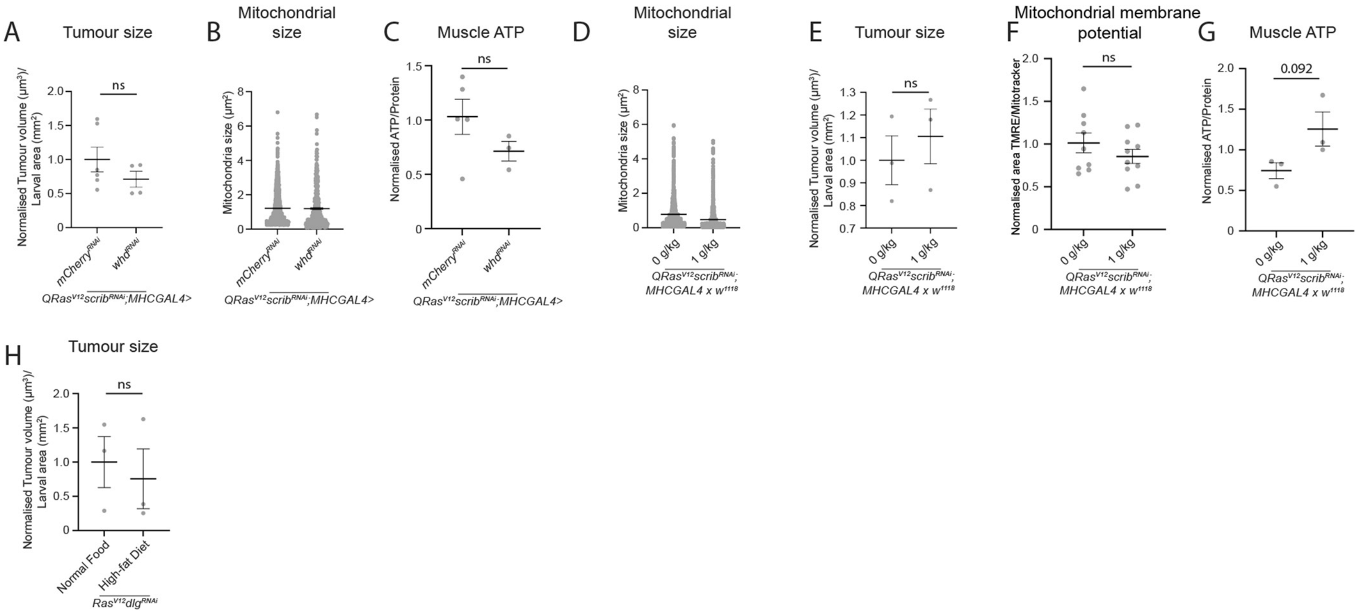

Figure EV5. Inhibition of Whd does not significantly affect mitochondria size, ATP levels or tumour size. Mitochondria size, tumour size, mitochondria membrane potential and ATP levels under dietary manipulations.

(**A**) Quantification of normalised tumour volume in $QRas^{V12}scrib^{RNAi}$;*MhcGAL4>mCherry*^{RNAi} and $QRas^{V12}scrib^{RNAi}$;*MhcGAL4> whd*^{RNAi} larvae (7 AEL), performed using Kruskal–Wallis as part of an analysis with EV2 A and EV3 F, which use the same controls ($n = 6$, 4). (**B**) Size distribution of individual mitochondria in the muscles of $QRas^{V12}scrib^{RNAi}$;*MhcGAL4>mCherry*^{RNAi} and $QRas^{V12}scrib^{RNAi}$;*MhcGAL4>whd*^{RNAi} larvae (6 AEL) ($n = 2037$, 550). (**C**) Quantification of normalised muscle ATP in $QRas^{V12}scrib^{RNAi}$;*MhcGAL4>mCherry*^{RNAi} and $QRas^{V12}scrib^{RNAi}$;*MhcGAL4> whd*^{RNAi} larvae (6 AEL), performed using one-way ANOVA as part of an analysis with EV2 C and EV3 E, which use the same controls ($n = 5$, 3). (**D**) Size distribution of individual mitochondria in the muscles of 7 days AEL $QRas^{V12}scrib^{RNAi}$;*MhcGAL4>mCherry*^{RNAi} larvae raised on a normal diet, or a diet containing 1 g/kg nicotinamide (NAM) ($n = 1447$, 1454). (**E**) Quantification of normalised tumour volume of 7 AEL $QRas^{V12}scrib^{RNAi}$;*MhcGAL4 >* w^{1118} larvae raised on a normal diet, or a diet containing 1 g/kg NAM, performed using Student's *t* test ($n = 3$, 3). (**F**) Quantification of the percentage of total mitochondria stained with MitoTracker™ Green that are shown to be active via TMRE incorporation in the muscles of 6 days AEL $QRas^{V12}scrib^{RNAi}$;*MhcGAL4>mCherry*^{RNAi} larvae raised on a normal diet, or a diet containing 1 g/kg NAM, performed using Student's *t* test ($n = 9$, 10). (**G**) Quantification of normalised muscle ATP in the muscles of 6 days AEL $QRas^{V12}scrib^{RNAi}$;*MhcGAL4>mCherry*^{RNAi} larvae raised on a normal diet, or a diet containing 1 g/kg NAM, performed using Student's *t* test ($n = 3$, 3). (**H**) Quantification of normalised tumour volume of $Ras^{V12}dlg1^{RNAi}$ larvae (8 AEL) raised on a normal diet, or a high-fat diet, performed using Student's *t* test ($n = 3$, 3). Data information: All error bars are +/− SEM. *P* values are: ns (not significant). Source data are available online for this figure.

 © The Author(s)
